# Loss of two-pore channel 2 enhances CD8+ T cell cytotoxicity and directly impairs tumour growth via MAPK axis in HCC

**DOI:** 10.3389/fimmu.2025.1668066

**Published:** 2025-10-24

**Authors:** Lina Ouologuem, Anna Kübler, Sarah Ouologuem, Amar Hadzic, Jan B. Stöckl, Anna Chiara Siciliano, Stefania Forciniti, Salvatore Nigro, Helena Iuele, Valentina Onesto, Anny Nguyen, Dana Matzek, Carla Abrahamian, Christian Grimm, Bastian Popper, Giuseppe Gigli, Loretta L. del Mercato, Olivia M. Merkel, Thomas Fröhlich, Sebastian Theurich, Karin Bartel

**Affiliations:** ^1^ Department of Pharmacy, Ludwig-Maximilians-Universität München, Munich, Germany; ^2^ Institute of Informatics, Ludwig-Maximilians-Universität München, Munich, Germany; ^3^ Gene Center, Cancer- and Immunometabolism Research Group, Ludwig-Maximilians-Universität München, Munich, Germany; ^4^ Department of Medicine III, Ludwig-Maximilians University Munich (LMU) University Hospital, Ludwig-Maximilians-Universität München, Munich, Germany; ^5^ German Cancer Consortium (DKTK), Munich Site, and German Cancer Research Center, Heidelberg, Germany; ^6^ Gene Center, Laboratory for Functional Genome Analysis, Ludwig-Maximilians-Universität München, Munich, Germany; ^7^ Institute of Nanotechnology – (NANOTEC, Consiglio Nazionale delle Ricerche (CNR), Lecce, Italy; ^8^ Department of Pharmacy, Pharmaceutical Technology, Ludwig-Maximilians-Universität München, Munich, Germany; ^9^ Biomedical Center, Core Facility Animal Models, Faculty of Medicine, Ludwig-Maximilians-Universität München, Munich, Germany; ^10^ Walther-Straub-Institute of Pharmacology and Toxicology, Ludwig-Maximilians-Universität München, Munich, Germany; ^11^ Department of Cardiology, German Heart Centre, TUM University Hospital, TUM School of Medicine and Health, Technical University of Munich, Munich, Germany; ^12^ German Centre for Cardiovascular Research (DZHK e.V.), partner site Munich Heart Alliance, Munich, Germany; ^13^ Department of Pharmacology, Faculty of Medicine, University of Oxford, Oxford, United Kingdom; ^14^ Immunology, Infection and Pandemic Research IIP, Fraunhofer Institute for Translational Medicine and Pharmacology ITMP, Munich/Frankfurt, Germany; ^15^ Cluster for Nucleic Acid Therapeutics Munich (CNAT-M), Munich, Germany

**Keywords:** lysosomal ion channels, HCC, immune evasion, TPC2, cancer, MAPK signalling, immune checkpoint

## Abstract

**Introduction:**

Hepatocellular carcinoma (HCC) remains a major global health challenge, characterised by limited therapeutic options and high mortality rates. Despite significant progress in systemic and immune-based therapies, many patients develop resistance or fail to respond, highlighting the need for new molecular targets. Lysosomal ion channels have recently emerged as important regulators of cancer biology; however, their involvement in tumour–immune interactions is still poorly understood.

**Methods:**

To investigate the role of the endolysosomal two-pore channel 2 (TPC2) in HCC, we employed genetic and pharmacological approaches, including TPC2 knockout (KO) and pharmacological inhibition using SG094. Functional analyses combining co-culture assays with CD8⁺ T cells, flow cytometry, and multi-omics profiling were conducted to assess the impact of TPC2 modulation on immune regulation, metabolic reprogramming, and intracellular signalling. Combination studies using SG094 and the immune checkpoint inhibitor Nivolumab were performed in vitro to evaluate synergistic effects.

**Results:**

Loss or inhibition of TPC2 enhanced CD8⁺ T cell-mediated cytotoxicity by increasing MHC-I and reducing PD-L1 expression both in vitro and in vivo. Combined treatment with SG094 and Nivolumab further augmented CD8⁺ T cell cytotoxicity compared with single-agent immune checkpoint blockade. Multi-omics analysis revealed that TPC2 KO disrupted amino acid metabolism, glycolysis, and protein translation, resulting in reduced ERK1/2 expression and impaired MAPK signalling. These metabolic and signalling alterations were associated with decreased tumour proliferation and increased MHC-I surface expression.

**Discussion:**

Our findings identify TPC2 as a dual regulator of tumour-intrinsic signalling and immune evasion in HCC. By modulating oncogenic MAPK activity and antigen presentation pathways, TPC2 influences both cancer progression and responsiveness to immunotherapy. Targeting TPC2 therefore represents a promising strategy to enhance immune checkpoint inhibitor efficacy in hepatocellular carcinoma.

## Graphical Abstract

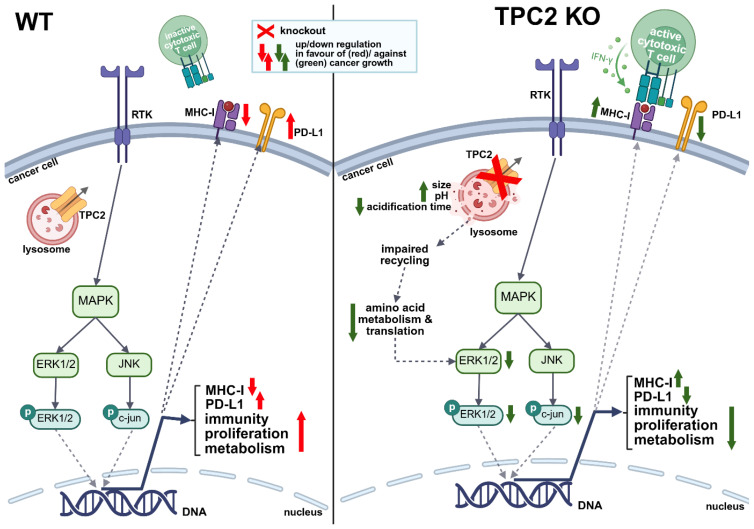

## 1 Introduction

HCC is the most common form of primary liver cancer, accounting for approximately 90% of all cases. It remains one of the leading causes of cancer-related mortality worldwide, with an estimated 850,000 new cases diagnosed annually (1). Well-established risk factors include chronic infection with hepatitis B or C viruses, excessive alcohol consumption, and dietary exposure to aflatoxin B1 (2). More recently, the increasing incidence of non-alcoholic steatohepatitis (NASH) has emerged as a major contributor to HCC development, particularly in Western countries (3).

Despite advances in the molecular characterization of HCC, many of the most prevalent genetic alterations, including mutations in TP53, CTNNB1, and AXIN1, remain challenging to target therapeutically (4). Consequently, systemic treatment options are limited. First-line therapies have traditionally included multikinase inhibitors such as sorafenib and lenvatinib, while second-line options comprise regorafenib, cabozantinib, and ramucirumab (5). Immune checkpoint inhibitors have recently transformed HCC treatment, with the combination of atezolizumab and bevacizumab demonstrating improved survival outcomes and receiving FDA and EMA approval (5). However, response rates remain suboptimal, and many patients develop resistance. This highlights the urgent need to identify novel and druggable targets to improve treatment efficacy in HCC.

In recent research the endolysosomal system (ES) emerges as key cellular compartment by which cells sustain multiple cancer hallmarks (6). Among the central lysosomal regulators, TPC2, a Ca²^+^- and Na^+^-permeable cation channel, has been increasingly recognised as a multifaceted contributor to tumour progression, including cancer proliferation, metabolism, migration, and angiogenesis (7–12). Lately, we identified GTPase Rab7 as an enhancer of TPC2 channel activity in melanoma, providing first-line evidence for a Rab7/TPC2/Wnt signalling axis that drives tumour growth, migration, and dissemination (12). TPC2 also regulates cell motility and vascularisation. Nguyen et al. identified TPC2 as a modulator of β1-integrin recycling, affecting migration and invasion (8), while Favia et al. showed that TPC2 promotes VEGF-induced angiogenesis via a VEGFR2/NAADP/TPC2/Ca²^+^ pathway *in vitro* and *in vivo* (9, 10). Complementary to this and most importantly, our lab has also recognized a reduction in cancer proliferation after loss of TPC2 function in HCC models (12, 13).

Beyond its tumour-intrinsic functions members of the TPC family may also influence immune evasion mechanisms in cancer (14). TPC2, for example, regulates inflammatory leukocyte recruitment and adhesion through P-selectin trafficking (15, 16) and plays a role in phagosome scission from the plasma membrane (17). TPC2-mediated Ca²^+^ release has been shown to support T cell cytolytic exocytosis, while TPC1 regulates histamine secretion in mast cells *in vivo* and *ex vivo* (18, 19). Moreover, lysosomal degradation of MHC-I, a process in which TPC2 may be involved, has been associated with reduced CD8^+^ T cell recognition and diminished immunotherapy efficacy (20, 21). Lysosomal pathways, including those influenced by TPC2, also modulate immune checkpoint expression such as CTLA-4 and PD-L1 (22, 23). In HCC, PD-L1 overexpression poses a therapeutic challenge due to its contribution to immune evasion within an immunosuppressive tumour microenvironment (24). Despite the clinical availability of immune checkpoint inhibitors (ICIs), many patients exhibit primary or acquired resistance (25, 26). Altered PD-L1 regulation, impaired antigen presentation, and immune-excluded phenotypes are key barriers to effective immunotherapy. Identifying regulators of PD-L1 trafficking and degradation, particularly within the lysosomal compartment (23, 27), may therefore offer new opportunities to enhance ICI responsiveness.

While the immunomodulatory functions of TPC2 have been increasingly recognised in melanoma, its role in HCC remains largely uncharacterised. Moreover, it is still unclear whether TPC2 or the related lysosomal calcium channel TRPML1 serves as the dominant regulator of tumour proliferation, metabolism, and immune evasion. Understanding which of these ion channels exerts primary control over lysosome-dependent cancer pathways is essential for identifying more selective and effective therapeutic targets. This study addresses this critical gap by systematically investigating the role of TPC2 in HCC, with a particular focus on its contribution to immune evasion, tumour growth, and metabolic reprogramming. In doing so, we relate our findings to existing data in melanoma and include a comparative analysis with TRPML1, confirming that the phenotypes observed are TPC2-specific and not the result of general lysosomal calcium flux disruption.

## 2 Methods

### 2.1 Cell lines and culture

RIL175 cells were provided by Prof. Simon Rothenfußer (CIPS-M, LMU Munich, Germany, 2016) (28). RIL175 TPC2 and TRPML1 KO cells were generated by our group (13, 29). Hep3B cells were obtained from the Leibniz Institute (ACC 93, 2015), and KOs were generated as described in this method section. HepG2 were purchased from DSMZ (German Collection for Microorganisms and Cell cultures) and Huh-7 cells from Japanese Collection of Research Biorescources (JCRB). B16F10luc cells were obtained from ATCC (CRL-6475-LUC2, 2022) and TPC2 and TRPML1 KO cells were generated as previously described (12, 30). SkMel5 WT and KO cells were provided by Prof. Christian Grimm (Walter-Straub-Institute of Pharmacology and Toxicology, LMU Munich, Germany, 2018). Jurkat cells were obtained from ATCC (TIB-152, 2017). RIL175, Huh7, HepG2 and SkMel5 were cultivated in Dulbecco’s modified Eagle’s medium (DMEM) (Anprotec, #AC-LM-0012) supplemented with 10% fetal calf serum (FCS) (Anprotec, #AC-SM-0027) at 37 °C, 5% CO_2_. For B16F10luc, Jurkat and Hep3B Roswell Park Memorial Institute (RPMI) 1640 medium (PAN-Biotech, P04-18500) supplemented with 10% FCS was used. None of the cell lines used are listed in the database of commonly misidentified cell lines maintained by ICLAC. All cell lines are proven to be mycoplasma-free quarterly via MycoSEQ™ (Eurofins, Munich, latest May 2025).

### 2.2 Generation of TPC2 and TRPML1 CRISPR/Cas9 knock out cell lines

The TPC2 and TRPML1 KO in Hep3B cells was conducted with the CRISPR/Cas9 system as described earlier (31). To do so, we deleted exon 2 of the TPCN2 or MCOLN1 gene. Single guide RNAs (sgRNAs) (**Supplementary Table S2**) were cloned into the pSpCas9(BB)-2A-GFP (PX458) plasmid (Addgene, #48138). The plasmids were transformed into competent DH5α-E.coli and subsequently prepared using the QIAGEN Plasmid Maxiprep Kit according to the manufacturer’s instructions. We then confirmed correct insertions by sequencing from the U6 promotor. After successful confirmation, Hep3B WT cells were transfected with both plasmids according to the Lipofectamine™ 3000 (Invitrogen) manufacturer’s instructions followed by single cell sorting (Cell Sorter BD FACSAria Fusion) into 96-well plates and subsequent clonal expansion. Successful exon 2 deletion was confirmed by standard PCR (Thermo Scientific Phusion Green Hot Start II High-Fidelity Polymerase, Thermo Fisher), agarose gel analysis and Sanger sequencing.

### 2.3 siRNA knockdown

TPC2 and TRPML1 siRNA knockdowns were generated to investigate the cellular differences human HCC cell lines Huh7 and HepG2. 0.5 Mio cells/well were seeded in a 6-well plate in their culture medium 24 h prior to transfection. On the day of transfection, cells were washed once with sterile PBS and the culture medium was replaced with 2 mL of prewarmed OptiMEM-medium just before the addition of the siRNA-lipid complexes. siRNA-lipid complexes were prepared according to the Thermofisher manufactor’s protocol using twice the amount of the transfection reagent Lipofectamine™ RNAiMAX (Cat#: 13778-100) and siRNAs at a total final concentration of 20 nM (non-targeted siRNA, TPCN2 (h) siRNA, or MCOLN-1 (h) siRNA). 4h after addition of the complexes OptiMEM medium was replaced with growth medium and cells were left for incubation for 48 h. Knockdown efficiency was determined via RT-qPCR. GAPDH served as a housekeeping gene.

### 2.4 Animal experiments

All animal experiments were approved by the District Government of Upper Bavaria, in accordance with institutional guidelines and the German Animal Welfare. Mice were kept in a temperature-controlled facility with a usual 12 h/12 h light and dark schedule. Humidity was kept at 60% and the room temperature at 22 °C, both of which were continuously monitored by operating technology. Light intensity in the facility was kept at around 120 lux and in the racks at around 40–60 lux. Mice were maintained in a group of four/five in cages that can be ventilated individually, and which had an area of 700 cm^2^ (IVC, Type 2 long, System Techniplast). In total, 48 C57Bl/6-Tyr mice (Envigo), female, 5 weeks old, were used for intravenous injection of 2,000,000 B16F10luc/RIL175 WT, TPC2 KO, or TRPML1 KO cells subcutaneously into the right flank. For tumour dissemination assay a total of 20 C57Bl/6-Tyr mice (Envigo) were injected with 200,000 RIL175 WT or TPC2 KO cells into the tail vein. Bioluminescence images were conducted using the IVIS Lumina system (PerkinElmer) on day 2, 5, 7, 9, 12 and 14 after intraperitoneal injection of 6 mg/mL luciferin per mouse. The tumour signal per defined region of interest was calculated as photons/second/cm (total flux/area) using the Living Image 4.4 software (Perkin Elmer). *In vivo* experiments were terminated on day 14 through and tumours were dissected, washed, photographed and weight. One half of the tumour was snap frozen in liquid nitrogen; the other half was fixed in 4% PFA. Animals were used under animal protocols approved by the government (Regierung von Oberbayern, ROB-55.2-2532.Vet_02-22-5, ROB-5.1-231 5682/LMU/BMC/CAM), and University of Munich (LMU) Institutional Animal Care Guidelines.

### 2.5 Immunohistochemistry

Tumour halves were fixed in 4% PFA (5 days, 4 °C), 1% PFA (2 days, 4 °C) and 70% EtOH (2 days, 4 °C) before proceeding with paraffin embedding. The tumours were then embedded in paraffin using an embedding centre (TN1700, Tanner Scientific). Paraffin-embedded tumours were sectioned into 10 µm thick slices using a microtome (HM355, Thermo Scientific). Duplicate sections were mounted onto glass slides.

#### 2.5.1 H&E staining

For H&E staining tissue sections were deparaffinized and rehydrated through a series of xylene and ethanol washes according to the manufacturer’s protocol (abcam, H&E staining kit, ab245880). Sections were stained in Mayer’s Haematoxylin solution for 30 seconds, dipped in Bluing agent and counterstained in Eosin solution for 1 min. Before sealing slides where washed and dehydrated through a series of dips in ethanol and finally xylene.

#### 2.5.2 DAB staining

For staining tissue sections were deparaffinized and rehydrated through a series of xylene and ethanol washes. Antigen retrieval was performed by incubating the sections in citrate buffer (pH 6.0) at 95 °C for 10 min. Endogenous peroxidase activity was quenched by treating the sections with 7.5% hydrogen peroxide for 10 min at RT. Primary antibodies were diluted accordingly in SignalStain^®^ Antibody Diluent (Cell Signaling Technology (CST), #8112S) and applied overnight at 4 °C: anti-CD8α (CST, #98941S, diluted 1:800), anti-PD-L1 (Cell Signaling Technology, #64988T, diluted 1:200), anti-MHC Class I (CST, #76828S, 1:250). The following day, sections were washed with phosphate-buffered saline (PBS, Sigma Aldrich, D8537) and incubated with SignalStain^®^ Boost Detection Reagent (HRP, rabbit) (CST, #8114P) for 30 min at RT. After final washing steps, SignalStain^®^ DAB Substrate Kit (CST, # 8059P) was added to each section and monitored closely until acceptable staining was obtained. Subsequently slides were immersed in water and stained with Hematoxylin (abcam, ab245880) for 2 min. After rinsing with water for 10 min tissue sections were mounted using FluorSave™ mounting medium (Merck, #345789) and coverslipped. Imaging was performed using an EVOS M5000 microscope (Thermo Fisher Scientific). Quantification and evaluation of PD-L1 and MHC-I stained tissue was conducted using the IHC profiler plugin in ImageJ according to Varghese et al. (32). Quantification of CD8^+^ positive cells was conducted using QuPath software (version 0.4.4).

### 2.6 Proliferation assays

#### 2.6.1 CellTiter-Blue assay

CellTiter-Blue assay was performed to determine cell proliferation of WT and KO cells. Therefore, 2,000 cells/well (RIL175, B16F10luc) or 5,000 cells/well (Hep3B, Skmel5) were seeded into a 96-well plate each using a multichannel pipette. Cells were left for incubation for 24h time periods at 37 °C (5% CO2). For measurement CellTiter-Blue reagent (Promega, G8080) was added in a 1:5 ratio. Cells were left for incubation for two hours at 37 °C (5% CO_2_). Then, fluorescence was measured by using the Tecan Spark^®^ Multimode Microplate Reader (Tecan, Männendorf, Switzerland) at 550 nm excitation and 595 nm emission wavelength. Relative proliferation was calculated the following way:


$$rel.\;proliferation=\;\frac{x-zero\;value}{Ctrl-zero\;value}$$

#### 2.6.2 Impendence measurement

Additionally, we used the xCELLigence RTCA device (ACEA Biosciences, San Diego, United States) to determine the doubling time and the cell index, a dimensionless parameter that is proportional to the cell number. Both can be determined through impedance measurement. Therefore, Hep3B, RIL175, Skmel5 or B16F10luc WT and KO cells were seeded into an equilibrated 16-well E-plate. Slopes were calculated using the xCELLigence RTCA software (ACEA Biosciences) for each cell line until reaching the plateau phase using equation:


$$Slope\;\left(\frac{1}{h}\right)=\;\frac{cell\;index-intercept}{time\;\left(h\right)}$$

### 2.7 Colony formation assay

Cells were seeded at a density of 2,000 cells/well (RIL175, B16F10luc) or 5,000 cells/well (Hep3B, Skmel5) of a 6-well plate and incubated for 7 days. Cells were fixed (4% PFA, 10 min, RT) and stained with crystal violet solution for 10 min at RT. Excess crystal violet solution(Sigma Aldrich, V5265-500ML) was removed with water and the plate was dried overnight. Subsequently, plates were imaged using the ChemiDoc™ Touch Imaging System (Bio-Rad) and colony size and formation was analysed using ImageJ by converting images to 8-bit, adjusting the threshold, and creating a selection to calculate the colony size and amount.

### 2.8 Confocal images

All confocal images were collected on a Leica DMi8 inverted scanning microscope (Leica, Wetzlar, Germany). Cells were grown in collagen-coated 8-well µ-slides (ibidi) overnight, fixed (4% PFA, 10 min, RT) and permeabilized (0.5% Triton-X in PBS, 10 min, RT; no permeabilization for MHC-1 and PD-L1 staining). Unspecific binding sites were blocked with 1% BSA in PBS (2 h, RT). After incubation with primary antibodies (overnight, 4 °C) and secondary antibodies (1 h, RT), cells were washed, mounted with FluorSave™ mounting medium (Merck Millipore, #345789), covered with glass cover slips, and imaged. Nuclei were stained with Hoechst 33342 (Sigma Aldrich, H3570). For LysoTracker stainings, cells were stimulated with LysoTracker Red (200µM, Thermo Fisher Scientific, L7528) and Hoechst 3342 for 30 min at RT, subsequently fixed (4% PFA, 10 min, RT), washed twice with PBS, mounted with FluorSave™ mounting medium (Merck Millipore, #345789) and imaged immediately.

### 2.9 pH-sensor measurements

To determine cell line specific stimulation conditions, the biocompatibility of the pH sensors was assessed via CellTiter-Glo assay prior to pH sensor measurements. Subsequently uptake efficiency in each cell line was quantitively analysed via flow cytometry. Therefore, cells were incubated with 0.05 mg/mL pH sensors for 0, 15, 30, 60, 120, and 360 minutes and their internalization were measured in all experimental conditions. Then, calibrations of pH sensor microparticles were performed at the beginning of each timelapse experiment for intracellular pH monitoring. To do this, pH sensors were exposed to different pH-adjusted cell media (range 4.0– 7.0) and acquired, along the z-axis, under controlled temperature (37 °C) and 5% CO_2_. After all preliminary assessments cells were seeded into an 8-well ibidi, left for incubation for 24h and exposed to pH-sensors. At different time points (0, 15, 30, 60 min) cells were fixed with PFA 4%, permeabilized with Triton X-100 0,1%, and incubated with Rabbit anti-LAMP1 (abcam, #278043) over night. Then, cells were incubated with Goat anti-rabbit 647 (Invitrogen, A21245) secondary antibody for 1h. Once the co-localization was confirmed, endo-lysosomal pH dynamics in each cell line was determined. Time-lapse experiments were conducted over 15 h via CLSM under controlled temperature and 5% CO_2_ to monitor cell uptake of the pH sensors and record their internalization over time. Computational analyses were applied to allow real-time tracking of pH sensor particles as previously described (33). The whole detailed methodology can be found in the **Supplementary Methods**.

### 2.10 Seahorse assay

Before cell seeding, the XFe96 microplate (Agilent Technologies) was coated with a collagen G solution (0.001% in PBS) for 30min at 37 °C 5% CO_2_. Subsequently, collagen G coating solution was removed, and wells were rinsed with sterile water. For comparison of WT and KO cells, cells were seeded at a density of 100,000 cells per well and allowed to settle for 4 h in assay medium without glucose (Agilent Technologies, 103575-100). The culture media were replaced with glucose-free assay media (DMEM, HEPS, pH7.4) supplemented with 2 mM glutamine and incubated for 1 h in a CO_2_-free incubator before measurement. The Seahorse XFe96 sensor cartridge was hydrated according to the manufacturer’s instructions. Extracellular flux analysis was performed as indicated by the manufacturer on a Seahorse XFe96 Analyzer (Seahorse Bioscience Inc., Santa Clara, CA, USA) according to the manufacturer’s instructions. Assays were performed according to Seahorse protocols with final concentrations of 5 mM glucose, 5 μM oligomycin, and 50 mM 2-deoxy-D-glucose (2-DG).

### 2.11 Western blot analysis

Cells were either treated as indicated (with SG-094 or TPC2-A1-P) or left untreated, trypsinized and washed twice with ice-cold PBS. Cells were lysed in detergent-containing buffer (1% NP-40, 0.1% SDS, 0.25% deoxycholate, 150 mM NaCl, 50 mM Tris-HCl in deionized water; pH 7.5) to obtain whole-cell lysates. Protease inhibitor cOmplete (Sigma Aldrich, 11697498001) was added directly before use. Protein content was analysed by Bradford assay against a BSA standard curve by measuring absorbance at 592 nm on a plate reader. Adequate amounts of 5x sample buffer (3.125 M Tris-HCl (pH 6.8), 50% glycerol, 5% SDS, 2% DTT, 0.025% Pyronin Y) and 1x sample buffer were added to adjust protein concentrations. Samples were applied to SDS-PAGE at 100 V for 21 min and subsequently at 200 V for 40 min. Successful protein loading was determined using stain-free technology and images were acquired on a ChemiDoc™ imaging system (Bio-Rad Laboratories, Hercules, CA, USA). The Trans-Blot Turbo™ (Bio-Rad) system was used to transfer proteins to 0.45µm NC membranes (Bio-Rad, #1620115). Membranes were washed with TBS-T and blocked with EveryBlot Blocking Buffer (Bio-Rad, #12010020),. Proteins were detected with specific primary antibodies, namely ERK1/2 (CST, #9102, 1:1000), pERK1/2 (Cell Signaling Technology (CST), #9106, 1:1000), JNK (Cell Signaling Technology (CST), #9252, 1:1000), pJNK (Cell Signaling Technology (CST), #9251, 1:1000), HLA class I (Cell Signaling Technology (CST), #76828, 1:1000), PD-L1 (Cell Signaling Technology (CST), #13684, 1:1000), c-jun (Cell Signaling Technology (CST), #9165, 1:1000), phosphor-c-jun (Cell Signaling Technology (CST), #3270, 1:1000) and corresponding HRP-coupled secondary antibodies using Clarity™ Western ECL Substrate (#1705060, Bio-Rad). Membranes were imaged using a ChemiDoc Touch imaging system (Bio-Rad). Data were processed in ImageLab (Bio-Rad), and protein band intensities were normalized to protein amount on the gel (stain-free detection). Full and uncropped blots are supplied as **Supplementary Data**.

### 2.12 Real-time quantitative PCR

Total RNA was extracted from cellular samples using the RNeasy Mini Kit (Qiagen, 73404) following the manufacturer’s protocol. After centrifugation, cells were washed twice with ice-cold PBS and resuspended in ice-cold RLT buffer supplemented with 40 µM DTT (R0861, Thermo Fisher). The concentration of the isolated mRNA was quantified using a Nanodrop Spectrophotometer. Subsequently, 2000 ng of RNA was reverse transcribed into cDNA using the High-Capacity cDNA Reverse Transcription Kit (Applied Biosystems, Waltham, MA, USA) in accordance with the supplier’s guidelines. Quantitative real-time PCR (RT-qPCR) was performed using PowerUp™ SYBR^®^ Green Master Mix (Applied Biosystems, A25776) in a reaction mixture containing 2 µl cDNA (equivalent to 50 ng), 6.25 µl PowerUp™ SYBR^®^ Green Master Mix, 3.25 µl RNase-free water, and 0.5 µl (200 nM) of both forward and reverse primers per well. The amplification reactions were carried out on the QuantStudio™ 3 Real-Time PCR System (Applied Biosystems), and relative gene expression levels were analysed using the ΔΔCT method (34). Actin was used as a housekeeping gene. Primers (**Supplementary Table S2**) were purchased from Metabion (Planegg, Germany) and validated for their specificity and efficiency prior to use.

### 2.13 Proteome analysis

Proteome analysis of RIL175 cells was performed as described in Frey & Ouologuem et al. (30). Hep3B and B16F10luc cells were lysed in 8 M Urea/50 mM NH_4_HCO_3_ in water using ultrasonication (Sonopuls GM3200, BR30, Bandelin, Berlin, Germany). Protein concentration was determined using a Pierce 660 nm assay (Thermo Fisher Scientific). After reduction and alkylation, samples were digested with LysC (1:100 enzyme:protein) for 4 h at 37 °C, and after dilution to 1M urea with 50 mM NH4HCO3 in water to 1 M Urea, trypsin was added (1:50 enzyme:protein) and samples were digested over night at 37 °C.

For mass spectrometry analysis a timsTOF HT mass spectrometer coupled with a nanoElute 2 LC system (both Bruker) was used. For each sample, 500 ng of peptides were injected and separated at 250 nL/min using Aurora Ultimate CSI (25 cm x 75 µm, IonOpticks) with the following eluents: 0.1% formic acid in water as eluent A and 0.1% formic acid in acetonitrile as eluent B. The separation method consisted of an initial ramp from 2% eluent B to 25% in 25 minutes followed by a 12 min gradient to 37%. MS spectra were acquired with the dia-PASEF mode (21–25 m/z wide windows and an ion mobility range of 0.85 and 1.27 1/k0). For protein identification, DIA-NN 1.9 (35) and the murine or human subset of the UniProtKB/Swiss-Prot database was used. Data analysis and statistical evaluation was performed using R.

For gene set enrichment analysis (GSEA), the label-free quantification data was log2 transformed, filtered for at least 4 valid values in at least one condition, and missing values were imputed from a normal distribution (width = 0.3; down shift = 1.8). The values were de-transformed and loaded into the GSEA and KEGG software ShinyGO 0.76 (36, 37). As a pathway database “GO Biological process” was selected, gene names were used without collapsing. The number of permutations was 10000.

### 2.14 RNA sequencing

To obtain total RNA cells were washed twice with ice-cold PBS and resuspended in ice-cold RLT buffer supplemented with 40 µM DTT (R0861, Thermo Fisher). Subsequently, RNA was extracted from cellular samples using the RNeasy Mini Kit (Qiagen) following the manufacturer’s protocol. The concentration of the isolated mRNA was quantified using a Nanodrop Spectrophotometer. RNA Sequencing was performed by Novogene (Planegg, Germany).

#### 2.14.1 RNA-seq alignment and read counting

The quality of the reads was checked using FastQC (v0.12.1) and MultiQC (v1.0dev0) (38). The reads were aligned using STAR (v2.7.10b) (39). First, the index was created with the parameter *sjdbOverhang* set to 149. Afterwards, the human data was aligned to genome GRCh38 (40) (ENSEMBL release 113). The mouse reads were aligned to GRCm39 (41) (ENSEMBL release 113). The reads were counted using featureCounts (v2.0.1).

#### 2.14.2 Differential gene expression

Differential Gene Expression Analysis was carried out using DESeq2 (42) (v1.44.0). Before running DESeq2, genes with a sum of less than 10 counts over all samples were filtered out. The resulting log-fold changes were shrunk using the *lfcShrink* function with the parameter *type* set to *apeglm.*

#### 2.14.3 Gene set enrichment analysis

Gene Set Enrichment Analysis was done using clusterProfiler (43)(v4.12.6). The log-fold changes from the Differential Gene Expression analysis were sorted decreasingly and given as input to the *gseGO* function. The parameter *OrgDb* was set to *org.Hs.eg.db*, *ont* to *BP*, *keyType* to *ENSEMBL*, and the *pvalueCutoff* to *0.05*.

### 2.15 Isolation of CD8 T cells from spleen

Spleens were obtained from C57Bl/6 mice and collected in ice-cold PBS immediately after mouse sacrifice. Single-cell suspensions of these organs were obtained using a gentleMACS dissociator (Miltenyi Biotec B.V.) and filtered through a 70 μm-pore cell strainer (Greiner, Bio-one). Single-cell suspensions were centrifuged (5 min, 500 g, 4 °C). Subsequently primary murine CD8^+^ T cells were isolated via magnetic bead separation with the CD8a^+^ isolation kit (130-104-075, Miltenyi Biotec B.V.) according to manufacturer’s protocol. After magnetic labelling and subsequent cell separation, cells were resumed in media, counted and seeded into a 6-well format (0.5Mio cells/mL). The purity of isolated CD8^+^ T cells was assessed by labelling with CD8-specific antibodies followed by flow cytometry analysis.

### 2.16 Co-culturing assay

Co-culture experiments were performed in 24-well plates without inserts. RIL175 cells (2 × 10^5^) or Hep3B cells (3 × 10^5^) were seeded in their respective growth media for 24 h. Jurkat cells were activated by adding T Cell TransAct™ (Miltenyi Biotec B.V., 130-111-160) and human recombinant IL-2 (Miltenyi Biotec B.V., 130-097-742) according to manufacturer’s protocol. Isolated CD8^+^ T cells were activated with T cell activation/expansion Kit, mouse according to manufacturer’s protocol (Miltenyi Biotec B.V., 130-093-627). After 24 h, media of cancer cells was changed to RPMI (+10% FCS) and murine CD8^+^ T cells or Jurkat cells were added directly to tumour cells in a 1:5 ratio. For combination therapy assay, cells were stimulated with 10µM SG094, 1.5 ng/mL Nivolumab and 40µg/mL Atezolizumab in the respective combinations 2 h after co-culturing. Tumour cells and T cells were left for co-cultivation for 24 h in total. Later, T cells were harvested for flow cytometry and media was harvested for Legendplex™ assay.

### 2.17 Flow cytometry

To determine lysosomal volume or pH, cells left untreated and loaded with 200 nM LysoTracker Red (Invitrogen, L7528) at 4 °C or with 1 µM LysoSensor Green (Invitrogen, L7535) at 37 °C for 30 min and subsequently washed and resuspended in ice-cold PBS. Fluorescence intensity LysoTracker Red was analysed using the PI channel, and fluorescence intensity of LysoSensor Green was analysed using the FITC channel. Before harvesting cells to investigate MHC-1, PD-L1, CD80 and CD86 surface levels cells were left untreated or treated with SG-094 and TPC2-A1-P as indicated. After harvesting, cells were washed with PBS twice, fixed with 4% PFA (10 min, RT), blocked with 1% BSA (10 min, 4 °C) and incubated antibody solution for 30 min at 4 °C in the dark. Respective antibodies: FITC anti-human CD274 (BioLegend, #393605), APC anti-human HLA-A,B,C (BioLegend, #311409), APC anti-mouse CD274 (BioLegend, #124311), PE anti-mouse H-2Db Antibody (Miltenyi Biotec B.V., #130-128-078). Subsequently cells, were washed with PBS twice and resuspended in PBS. All flow cytometry experiments named above were performed on a AttuneNxT (ThermoFischer). Data were evaluated using FlowJo 10.0.

### 2.18 Legendplex™ mouse inflammation panel (13-plex)

Supternatants from co-culturing experiments were analysed via Multiplex analysis via flow cytometry with the Legendplex™ Mouse Inflammation panel (13-plex) (BioLegend, 740150). Samples were prepared according to manufacturer’s protocol. Therefore, supernatant from the coculturing assay was harvested by centrifuging (10,000 rpm, 5 min, RT) and transferring to a new Eppendorf tube. Then, 25µL of Assay buffer was added to each well, subsequently 25µL of each standard or each sample was added to the assigned wells. After adding 25µL of mixed beads, the plate was sealed with a plate sealer, covered in aluminium foil and placed on a plate shaker (800 rpm, 2 h, RT). Then, the plate was centrifuged (250 g, 5 min, RT) and the supernatant discarded by flicking motion. Plate was washed once with 200µL washing buffer (800 rpm, 1 min, RT) and centrifugation step was repeated. Afterwards 25µL Detection Antibodies were added to each well, the plate sealed and covered in aluminium foil and placed on a plate shaker (800 rpm, 1 h, RT). 25µL SA-PE were directly added to each well, followed by an incubation time (800 rpm, 30 min, RT). After centrifugation and washing, 150µL of washing buffer was added and beads were resuspended by pipetting. Samples were directly measured on an AttuneNxT (ThermoFischer). Evaluation was done via LegendPlex Software (BioLegend, Version 2024-06-15).

### 2.19 Statistical analysis

All experiments were conducted at least three times independently and data represents means ± SD, unless stated otherwise. For quantification of images, at least 400 individual cells have been analysed per biological replicate using ImageJ. Unless stated otherwise, statistical significance was determined with an unpaired t-test with Welch’s correction or ordinary one-way ANOVA using GraphPad Prism 10.0, San Diego, USA. Results were considered significant for p< 0.05.

## 3 Results

### 3.1 TPC2 KO impairs lysosomal properties and acidification potential

To enable cross-species comparison, we established and characterised TPC2 and TRPML1 KO cell lines in the human hepatocellular carcinoma line Hep3B, complementing our existing murine RIL-175 TPC2 KO model (13, 30) (**Supplementary Figures S1A-C**). Given that both TPC2 and TRPML1 are lysosomal calcium channels of distinct families implicated in cancer progression, TRPML1 served as a comparative control to determine whether observed effects are unique to TPC2 or reflect a broader lysosomal calcium signalling phenomenon.

As TPC2 and TRPML1 primarily localise to lysosomes, we first sought to characterise how their deletion alters lysosomal structure and function, providing a mechanistic foundation for their downstream effects on cancer progression. While prior studies have linked TPC2 loss to increased lysosomal mass, its impact on lysosomal pH and acidification capacity is still being debated in the literature (12, 44, 45).

**Figure 1:**
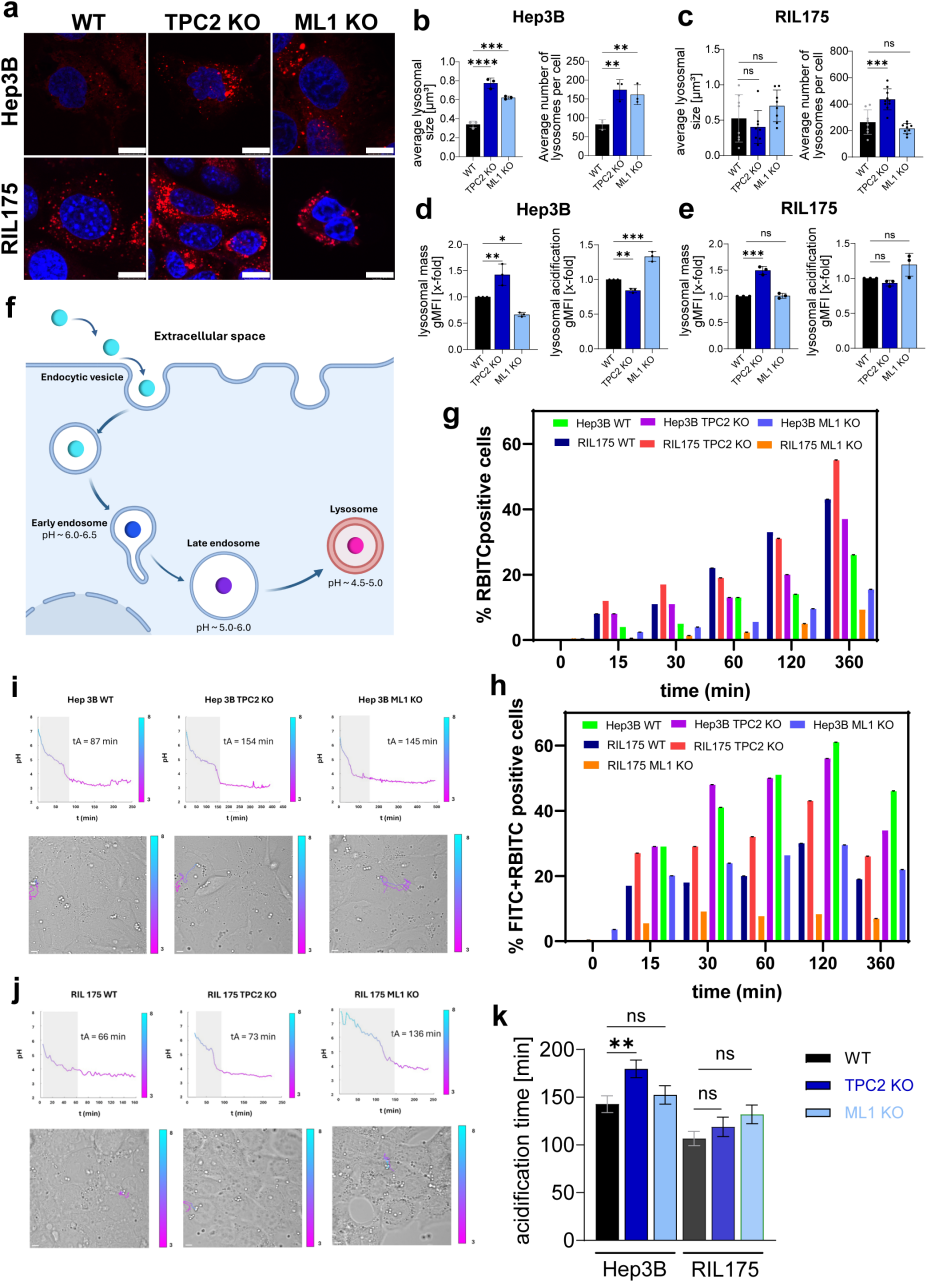
Characterisation of lysosomal properties after TPC2 KO. **(A)** LysoTracker Red stainings of Hep3B and RIL175 cells. Nuclei are shown in blue (Hoechst) (n=3). Representative images shown. Scale bar 10 µm. **(B, C)** Average lysosomal size and average number of lysosomes per cell from (a) were determined using colour threshold in ImageJ. **(D, E)** Lysosomal mass determined by LysoTracker Red staining and lysosomal acidification by LysoSensor Green staining for 2h, followed by subsequent flow cytometer analysis and evaluation of gMFI. All n=3. Statistical significance was assessed by One-way ANOVA. * p< 0.05, ** p< 0.01, *** p< 0.001, **** p<0.0001, ns = not significant. **(F)** Schematic illustration on pH sensor functionalities. **(G)** Quantification of RBITC-positive cells, indicating total sensor uptake, extracted from dot plot analysis in **(A)**. **(H)** Quantification of dual-positive cells (FITC^+^/RBITC^+^), reflecting uptake of intact, functional pH sensors. Values are expressed as mean ± SE of three independent experiments (n = 3). **(I, J)** Representative time-lapse tracking of individual pH sensor particles, illustrating dynamic changes in fluorescence corresponding to intracellular acidification over time. Scale bars: 5 μm. **(K)** Acidification times across different cell lines. Outliers were determined via ROUT Q=1%. Graph shows mean ± SEM. Statistical significance was evaluated using a two-tailed Wilcoxon−Mann−Whitney test.

In line with a previous study by our lab (44), TPC2 KO cells display significantly increased lysosomal size and mass across multiple cell lines (**Figures 1A-E**). Additionally, the number of lysosomes per cell was significantly elevated (**Figures 1B, C**). Further, TPC2 KO cells exhibited reduced lysosomal acidity compared to WT cells indicated by a decrease in LysoSensor Green signal, whereas TRPML1 KO cells showed enhanced acidification relative to WT and TPC2 KO cells (**Figures 1D, E**). Yet, LysoSensor Green measurements have certain limitations including non-ratiometric, non-quantitative pH detection, susceptibility to photobleaching and cytotoxicity, restriction to highly acidic compartments, and potential signal variability due to dye leakage or lysosomal volume changes (46–48).

Thus, to accurately assess whether TPC2 influences endo-lysosomal pH regulation, we synthesized ratiometric, silica-based optical pH sensors (**Figure 1F**) via a one-pot bottom-up approach, incorporating the pH-sensitive FITC probe and the reference dye RBITC (**Supplementary Figure S1D**) (49–52). These particles were uniform (**Supplementary Figure S1D**), showed excellent pH responsiveness and reversibility across cycles (**Supplementary Figure S1D**) and were biocompatible (**Supplementary Figure S1E**) confirming their suitability for intracellular pH monitoring (53). Notably, the percentage of RBITC-positive cells increases over time, indicating successful uptake of the pH sensors in each cell line (**Supplementary Figure S1G**). Specifically, Hep3B WT and KO cells display a higher uptake efficiency compared to RIL175 WT and KO cells (**Figure 1G**). Interestingly, within the same cell line, TPC2 KO cells (37% for Hep3B and 55% for RIL175) exhibit increased uptake compared to their WT counterparts (26% for Hep3B and 43% for RIL175) (**Figure 1G**), suggesting a potential link between TPC2 function and particle internalization dynamics. In contrast, ML1 KO cells do not display enhanced uptake compared to WT, suggesting that ML1 may play a less prominent or distinct role in the internalization process compared to TPC2.

Additionally, the percentages of both FITC- and RBITC-positive cells (**Figure 1H**) increase over time, reaching a peak at 120 minutes post-incubation before decreasing in each cell line, likely due to sensor confinement into progressively more acidic endosomal/lysosomal compartments, causing a decrease in FITC fluorescence intensity. Additionally, CLSM micrographs extrapolated from time-lapse experiments of 15 hours at the initial time (T_i_), intermediate time (T_int_), and final time (T_f_) for each cell line (54). Specifically, before internalization (T_i_), extracellular pH sensors display a strong yellow fluorescence due to the neutral pH of the cell medium. Notably, following the cell’s internalization, the pH sensors display a strong red fluorescence revealing confinement in intracellular acidic compartments (e.g., endosomes/lysosomes) (**Supplementary Figure S1H**).

Confocal imaging confirmed that internalized pH sensors co-localize with LAMP-1–positive lysosomes, validating their specificity for tracking lysosomal acidification (**Supplementary Figure S1I**). Time-lapse microscopy revealed distinct intracellular acidification kinetics between TPC2 WT and KO cells. Using z-stack acquisitions and prior sensor calibration (pH 4.0–7.0) (**Supplementary Figure S1G**), we quantified the FITC/RBITC fluorescence ratio over time, showing a slowed decrease in FITC signal in TPC2 KO cells, indicative of impaired lysosomal acidification (**Figures 1I-K**). A slower decrease in FITC signal was also observed in ML1 KO cells compared to WT cells, for both HEP3B (pvalue< 0.0001) and RIL175 (p=0.0004).

These findings demonstrate that TPC2 KO increases lysosome size and number while impairing acidity, whereas TRPML1 KO enhances acidification without consistent morphological changes. Notably, TPC2 KO significantly delays lysosomal acidification in Hep3B cells but not in RIL175 cells, highlighting a cell line-dependent effect. These findings support a role for TPC2 in regulating lysosomal Ca²^+^ and H^+^ homeostasis, with high-resolution pH sensor data confirming the robustness of these observations.

### 3.2 Knockout of TPC2 drastically reduces cancer cell growth *in vivo*

**Figure 2:**
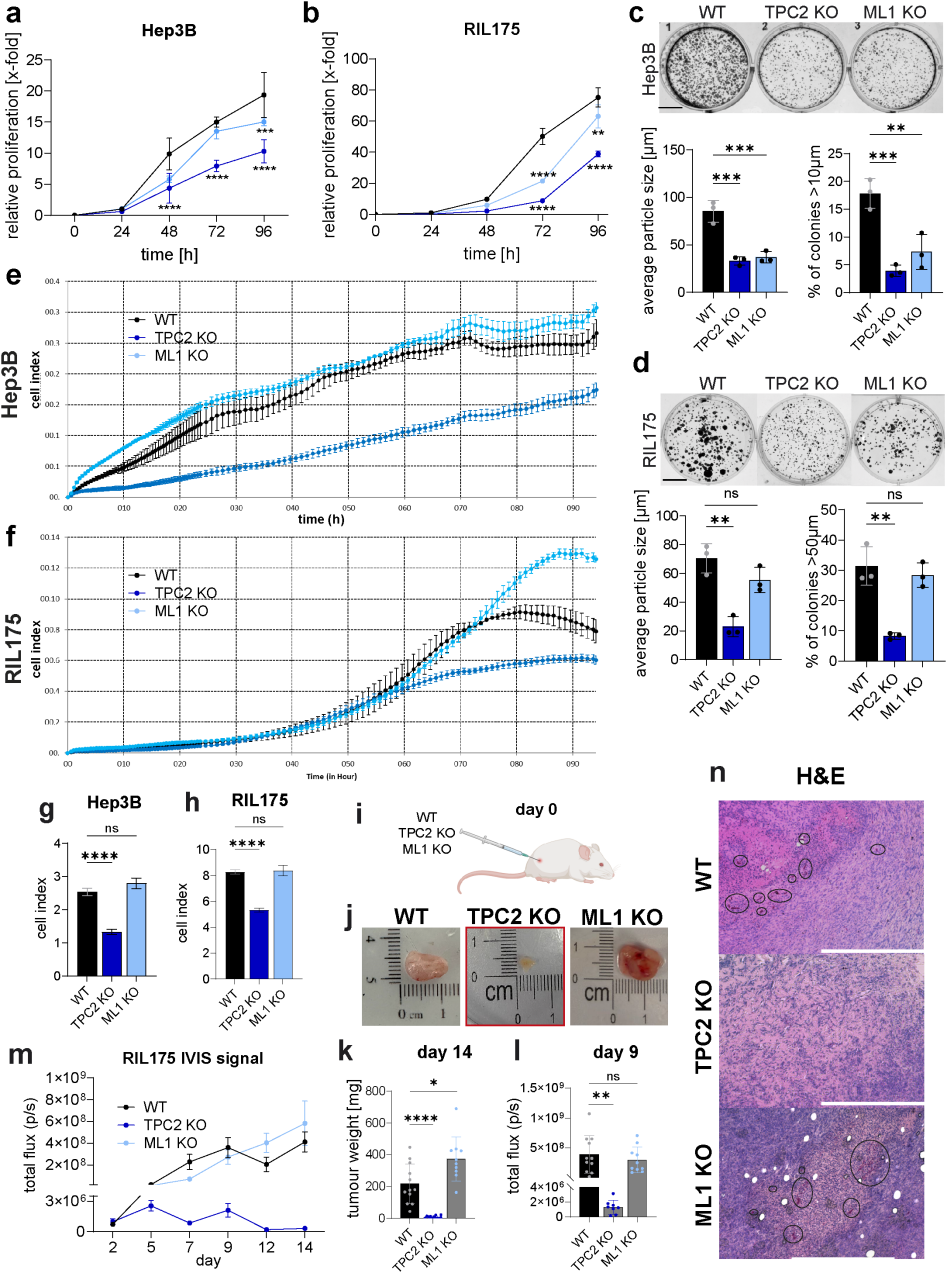
Loss of TPC2 reduces cancer cell proliferation *in vitro* and tumour growth *in vivo*. **(A, B)** KO of TPC2 leads to significantly reduced cancer cell proliferation monitored via CellTiter-Blue assay over 96h (n=5) **(C, D)** Colony formation in TPC2 KO cells significantly reduced over 5 days (n=3). Scale bar 1cm. **(E-H)** Impendence measurements to determine cell index and doubling time over 92h (n=3). **(E, F)** XCelligence impedance measurements over 92h. Cell index and calculated doubling time from impedance measurements of Hep3B **(G)** and RIL175 **(H)** cells. **(I)** Ectopic *in vivo* tumour model (n=12 each group) shows significantly reduced TPC2 KO tumours. **(J)** Images of *ex vivo* RIL175 tumours after endpoint day 14. Four mice from the RIL175 TPC2 cohort had no tumours left. **(K)** Tumour weights after endpoint day 14 show significant reduction in TPC2 KO tumours. One mouse in the RIL175 ML1 KO cohort had to be terminated early, due to sickness. **(L)** Total flux on day 9. **(M)** Tumour size was indirectly measured non-invasively via IVIS imaging over 14 days. Graphs show a significant reduction in tumour growth in TPC2 KO tumours. Outliers were identified with ROUT, Q=1%. **(N)** IHC of WT, TPC2 KO and ML1 KO tumours from ectopic HCC tumour model displaying more blood vessels in WT and ML1 KO tumours (black ellipses). Also, massive necrosis in TPC2 KO tumours, accompanied with elevated MHC-1 levels. Statistical significance was assessed by two-way ANOVA Dunnett’s multiple comparisons test (a-d) or one-way ANOVA Dunnett’s multiple comparisons test * p< 0.05, ** p< 0.01, *** p< 0.001, **** p<0.0001, ns, not significant.

We then sought to investigate the magnitude of proliferation impairment upon TPC2 deletion in our model. We show that loss of TPC2 function has a strong effect on cancer proliferation (**Figures 2A-B, E, F**, **S2A-F**) and colony formation (**Figures 2C, D**, **S2G, H**). Whereas TRPML1 KO cells displayed a cell line dependant inhibition of cell proliferation, in Hep3B cells TRPML1 KO reduced the ability to form colonies (**Figure 2C**), whilst in RIL175 TRPML1 KO retained their ability to form colonies like WT cells (**Figure 2D**).

The anti-proliferative effect was observed in metabolism based (**Figures 2A, B**, **S2A, B**), as well as impedance-based proliferation measurements (**Figures 2E-H**, **S2C-F**), while TRPML1 KO shows a minor effect in both methods.

In line with our *in vitro* data, tumour growth of TPC2 KO tumours was significantly impaired, whereas TRPML1 KO tumours showed no significant reduction compared to WT tumours in an ectopic HCC model (**Figures 2I-M**). Notably, four mice initially bearing TPC2 KO tumours, had no tumours left on day 14. Of note, TPC2 KO cells disseminated less in periphery organs compared to WT cells (**Supplementary Figures S2I, J**). In melanoma, we have already shown that TPC2 KO leads to reduced tumour growth (12), while we here show that TRPML1 KO did not show an effect on tumour progression in melanoma (**Supplementary Figures S2K-N**). Notably, a KO of TPC2 impacted tumour growth in melanoma less than in HCC, whilst four tumours went into remission in HCC none did in melanoma model. Also, average tumour weight of HCC tumours on day 14 was 7.658 g, whilst melanoma tumours were almost ten times heavier with 78.84 g (**Figures 2K**, **S2N**). These findings confirm that TPC2 deletion significantly impairs cancer cell proliferation and tumour growth, while TRPML1 KO has no significant effect *in vivo* and the effect was more profound in HCC than in melanoma. Further *ex vivo* tumour sections of TPC2 KO tumours displayed massive necrosis, whereas WT and TRPML1 KO tumours were characterised by high cell density and well-distributed blood vessels throughout the tumour (**Figure 2N**). Notably, the impact of TPC2 loss is strongly pronounced in HCC, raising the question of whether its role in tumour suppression extends beyond inhibiting proliferation alone.

### 3.3 TPC2 KO enhances CD8^+^ T cell infiltration and cytotoxicity

**Figure 3:**
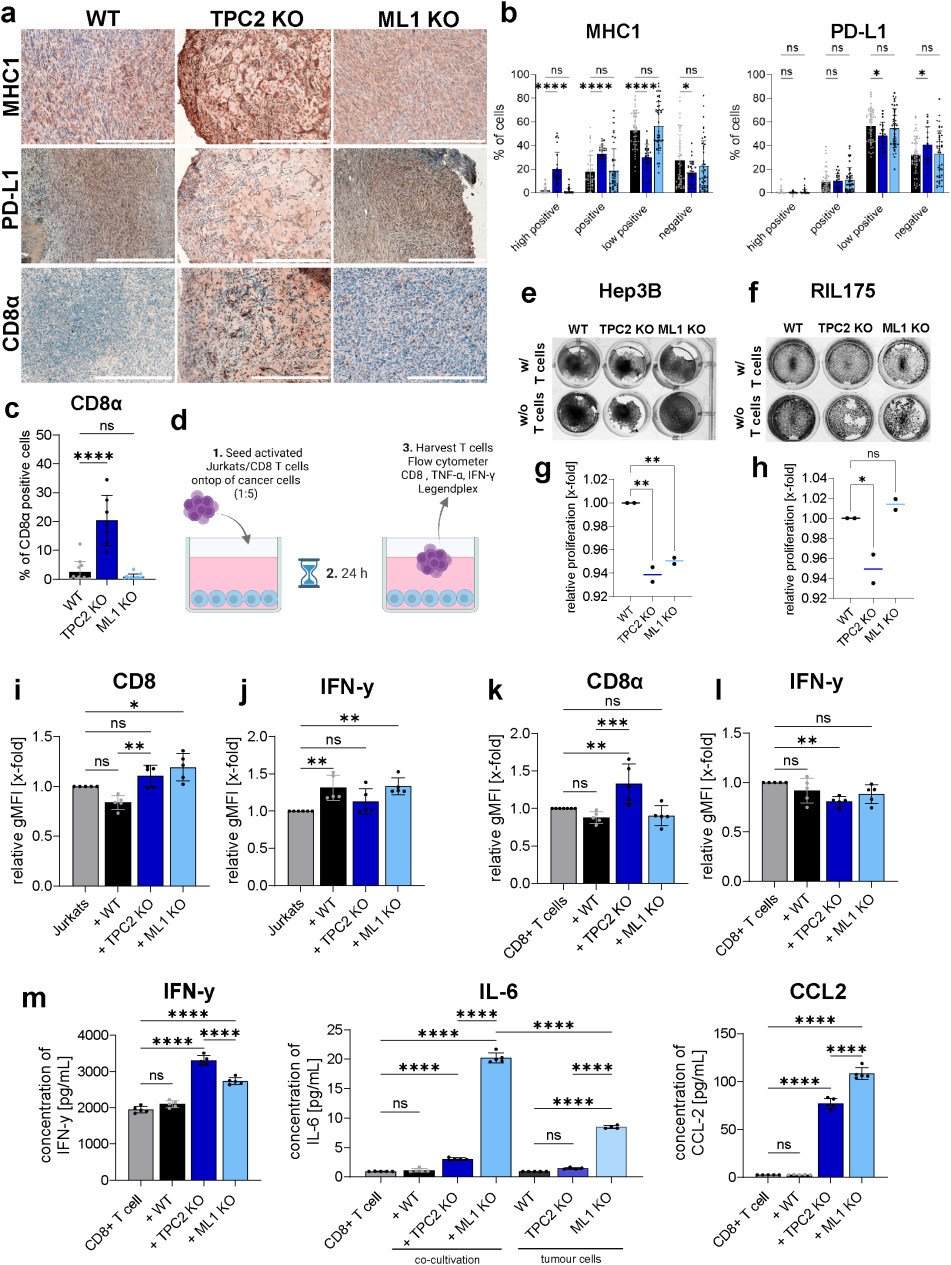
TPC2 KO sensitises cytotoxic CD8 T cells to tumour. **(A)** IHC staining of ex vivo tumours from ectopic *in vivo* experiment show elevated MHC1, **(B)** reduced PD-L1 levels **(B)** and higher CD8 infiltration in TPC2 KO tumours **(C)**. All tumours obtained from *in vivo* experiments (**Figure 2**) were stained and whole tumour was imaged and analysed. % of CD8 positive cells was determined using positive cell selection in QuPath (0.4.4.). Scale bar 300µm. **(D)** Co-culturing experiment served as an *in vitro* method to reproduce *in vivo* settings. After 24h co-cultivation with Jurkat cells or T cells **(E)** Hep3B and **(F)** RIL175 cells were stained with crystal violet. **(G, H)** Direct co-culturing experiments of T cells with Hep3B **(G)** or RIL175 **(H)** cells over 72h. Endpoint quantification via Cell-Titer Blue Assay. Jurkats **(I, J)** or murine CD8^+^ T cells **(K, L)** were analysed on extracellular CD8 and intracellular IFN-γ levels. T cells co-cultured with TPC2 KO cells display higher CD8 and lower IFN-γ levels. **(M)** Supernatant of RIL175/CD8^+^ T cell co-culture analysed with LegendPlex inflammation panel via flow cytometry. IFN-γ levels increased in TPC2-co-culture, whilst IL-6 and CCL2 significantly increased in ML1 KO setting. Statistical significance was assessed by two-way ANOVA Dunnett’s multiple comparisons test or one-way ANOVA Dunnett’s multiple comparisons test * p< 0.05, ** p< 0.01, *** p< 0.001, **** p<0.0001, ns, not significant.

To investigate whether TPC2 KO influences tumour immunogenicity and immune evasion mechanisms, we analysed key immune markers *in vivo*. On this note, immunohistochemistry (IHC) revealed that TPC2 KO tumours exhibited significantly elevated MHC-I levels and significantly reduced PD-L1 expression compared to WT tumours (**Figures 3A, B**). In contrast, TRPML1 KO tumours showed no significant differences in MHC-I or PD-L1 expression relative to WT tumours. Enhanced tumour cell recognition and elimination was confirmed by significantly higher CD8α T cell infiltration in TPC2 KO tumours, whereas TRPML1 KO tumours showed no significant difference compared to WT (**Figures 3A, C**).

Additionally, we conducted *in vitro* co-culture assays combining Hep3B cells with activated T cells and RIL175 cells with activated CD8^+^ T cells isolated from C57BL/6 mice (**Figure 3D**) to investigate changes in cytotoxicity of CD8 T cells. After 24h of co-culturing TPC2 KO cells showed less proliferation capacities compared to their control (**Figures 3E, F**). Quantification of relative cancer cell proliferation in co-culture with T cells confirmed these results as TPC2 KO cells showed reduced proliferation capacities compared to the respective WT cells after 72h (**Figures 3G, H**). Further T cells were analysed via flow cytometry (**Figures 3I, K**, **S3A-C**), which revealed increased CD8 surface expression in T cells co-cultured with TPC2 KO cells compared to WT controls (**Figures 3I, H**). Intracellular TNF-α levels remained unchanged across all conditions (**Supplementary Figures S3D, E**). Meanwhile intracellular IFN-γ levels were elevated in T cells co-cultured with WT and TRPML1 KO Hep3B cells (**Figure 3J**), whereas in the RIL175 system, IFN-γ levels were significantly reduced in TPC2 KO-associated T cells but unchanged in WT and TRPML1 KO condition (**Figure 3L**). These results suggest that TPC2 deletion not only impairs tumour cell proliferation but also reduces immune evasion by enhancing CD8^+^ T cell activation and cytotoxic potential. This underscores the role of TPC2 as a modulator of tumour immunogenicity, whilst TRPML1 shows no similar immunological effect.

To evaluate cytokine secretion, we performed multiplex analysis with the supernatants of the RIL175/CD8^+^ T cell co-cultures. Consistent with the intracellular data, TNF-α secretion remained unaltered (**Supplementary Figure S3F**). In contrast, IFN-γ secretion was significantly elevated in the TPC2 KO co-culture compared to WT indicating higher cytotoxicity. TRPML1 KO co-cultures showed no significant differences in IFN-γ levels but exhibited significantly increased IL-6 and CCL2 secretion compared to both WT and TPC2 KO conditions (**Figure 3M**), suggesting a pro-tumorigenic environment.

Together, our findings demonstrate that TPC2 inhibition enhances anti-tumour immunity by upregulating MHC-I expression, downregulating PD-L1, and promoting CD8^+^ T cell-mediated cytotoxicity. In contrast, TRPML1 knockout did not replicate these immune-stimulatory effects, underscoring the specific role of TPC2 in modulating tumour immune evasion.

### 3.4 TPC2 regulates MHC-I and PD-L1 surface expression on cancer cells

**Figure 4:**
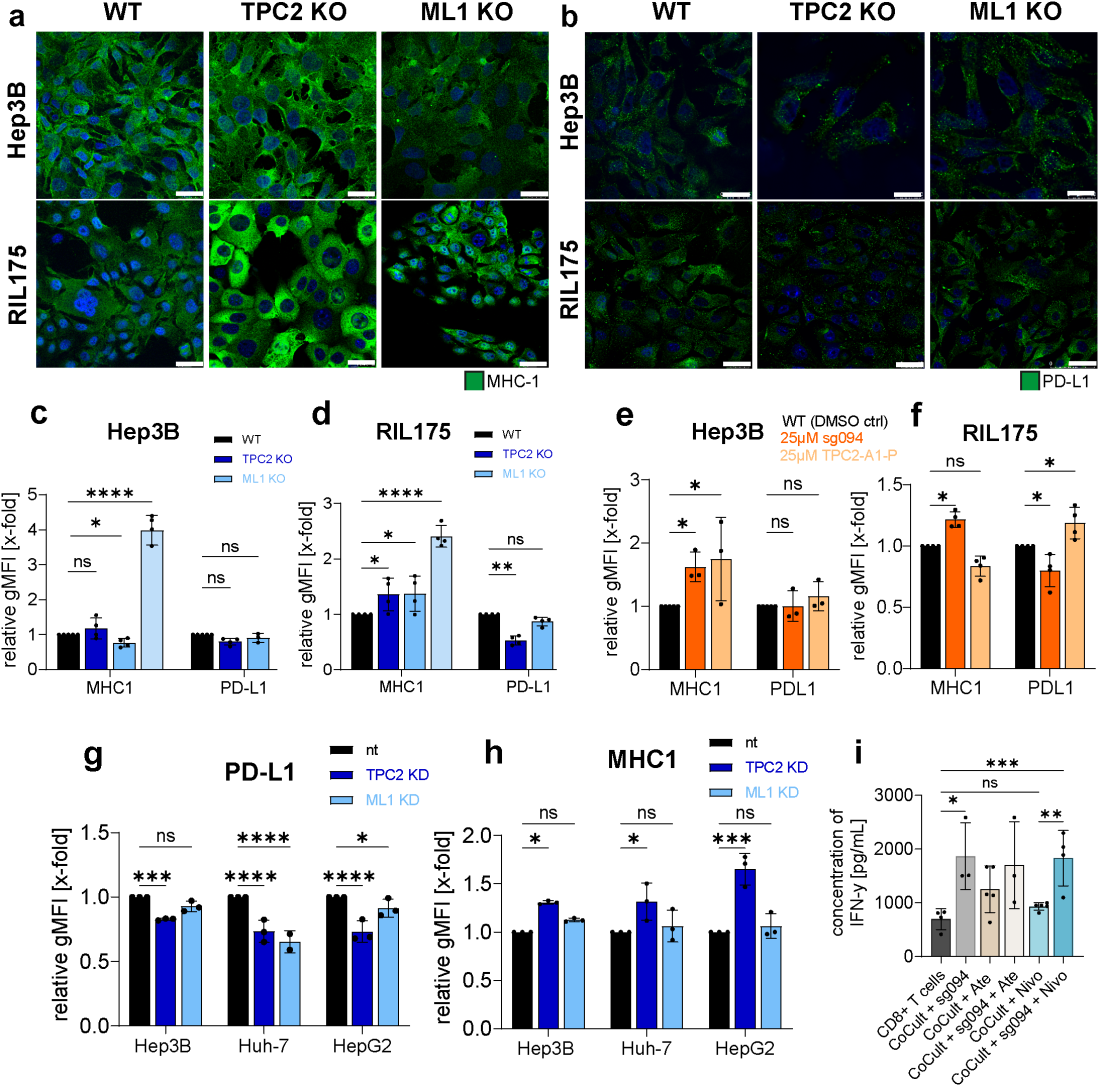
Loss of TPC2 function leads to elevated MHC-1 and reduced PD-L1 surface levels. **(A, B)** Confocal images of MHC-1 **(A)** or PD-L1 **(B)** on cancer cell surface (n=3). Cells were not lysed prior antibody addition. Treatment of cells with 100ng/µL IFNγ for 24h served as positive control. Representative images shown. Scale bar 10µm. **(C, D)** Flow cytometer analysis of MHC-1 and PD-L1 on cancer cell surface (n=5). Quantification resulted over evaluation of gMFIs. **(E, F)** MHC-1 and PD-L1 levels on cell surface of Hep3B and RIL175 cells after 24h treatment with 25µM SG-094 or 25µm TPC2-A1-P (Hep3B n=3, RIL175 n=4). **(G, H)** Flow cytometer analysis of MHC-1 and PD-L1 surface expression of non-targeted (nt), TPC2 KD and ML1 KD human HCC cell lines. **(I)** Supernatant of RIL175/CD8^+^ T cell co-culture treated with respective ICI (Ate= Atezolizumab, Nivo=Nivolumab) and/or SG094 analysed with LegendPlex inflammation panel on IFN-γ levels via flow cytometry. Data was conducted from five different co-culturing experiments. Statistical significance was assessed by One-way ANOVA Dunnett’s multiple comparisons test * p< 0.05, ** p< 0.01, *** p< 0.001, **** p<0.0001, ns = not significant.

To elucidate how the observed enhancement of anti-tumour immunity is related to TPC2, we assessed the impact of TPC2 loss on MHC-1 and PD-L1 expression levels *in vitro*. Confocal images and flow cytometry revealed that TPC2 KO consistently led to increased MHC-I expression (**Figures 4A, D**), essential for presenting tumour antigens to CD8^+^ T cells. IFN-γ served as a positive control significantly upregulating MHC-I levels in both Hep3B and RIL175 cells (**Figures 4C, D**). Additionally, PD-L1 expression, which usually leads to dampening of the anti-tumour T cell response, was diminished upon TPC2 KO in RIL175 cells (**Figures 4B, D**). In contrast, TRPML1 KO resulted in having minimal or inconsistent effects in other cell lines on MHC-I and PD-L1 levels (**Figures 4A-D**). These findings were confirmed by Western Blot analysis (**Supplementary Figures S4A, B**). To explore whether these findings extend to melanoma, we evaluated MHC-I and PD-L1 expression in two melanoma cell lines. Skmel5 TPC2 KO cells mirrored the HCC phenotype, showing elevated MHC-I and reduced PD-L1 expression (**Supplementary Figures S4C, D**), whereas B16F10luc cells remained largely unaffected (**Supplementary Figures S4C, E**), correlating with the more substantial tumour weight reduction observed in the HCC model compared to melanoma (**Supplementary Figures S2L, N**).

Treatment of Hep3B cells with TPC2 inhibitors or activators resulted in non–TPC2-specific effects. Meanwhile, in RIL175 cells the observed effects were directly linked to TPC2 function, as pharmacological TPC2 inhibition by SG094 mimicked the KO phenotype, leading to increased MHC-I and decreased PD-L1 expression *in vitro*, while TPC2 activation by TPC2-A1-P reversed these effects (**Figure 4F**). Consistent a siRNA knockdown of TPC2 resulted in the same phenotype in both human and murine HCC cells (**Figures 4G, H**). All changes in surface marker expression were quantified using flow cytometry.

To assess the impact of TPC2 inhibition on CD8^+^ T cell activation, we quantified IFNγ secretion under various treatment conditions. Pharmacological inhibition of TPC2 by SG094 significantly increased IFNγ production in CD8^+^ T cells, both in monoculture and in co-culture with murine HCC cells (**Figure 4I**). Notably, combination treatment with SG094 and the PD-1 inhibitor Nivolumab further enhanced IFNγ secretion compared to either treatment alone. Similarly, monotherapy with the PD-L1 inhibitor Atezolizumab elevated IFNγ levels, and this effect was sustained when combined with SG094 (**Figure 4I**).

Along the line, expression of co-stimulatory molecules CD80 and CD86, which are essential for efficient T cell activation (55), remained stable in all but RIL175 TPC2 KO cells. In contrary, TRPML1 KO significantly reduced CD80 and CD86 expression across cell lines, (**Supplementary Figures S4F, G**), suggesting differential effects of lysosomal calcium channels on immune modulation.

In summary, TPC2 loss not only suppresses proliferative signalling but also enhances tumour immunogenicity, immunogenicity by an increase in MHC-1 expression and decrease in PD-L1 expression, particularly in HCC. Pharmacological targeting of TPC2 with SG094 increased IFNγ secretion in CD8^+^ T cells, both alone and in tumour co-culture settings, and further elevated cytokine release when combined with immune checkpoint inhibitors. These results highlight a synergistic potential between TPC2 inhibition and checkpoint blockade. These findings suggest a dual, tumour-type–specific role for TPC2 in promoting cancer progression through immune escape mechanisms.

### 3.5 Multi-omics profiling reveals TPC2 as a key regulator of tumour cell metabolism and proliferation

In order to uncover the molecular mechanisms underlying the phenotypic differences between WT, TPC2 KO, and TRPML1 KO cells, we applied an unbiased, integrative multi-omics approach, including transcriptomic and proteomic profiling, followed by principal component analysis (PCA), differential gene expression analysis, and functional enrichment.

**Figure 5:**
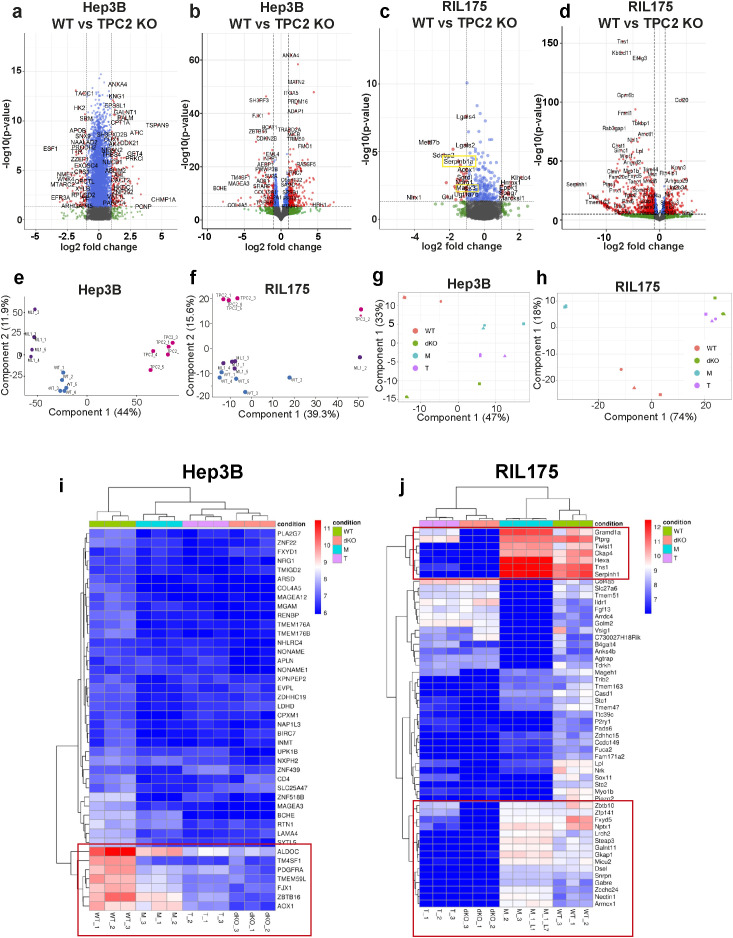
Proteomic and RNA-sequencing data. **(A, B)** Volcano plots of Hep3B WT vs TPC2 KO proteomic **(A)** and RNA-seq **(B)** data displaying all differentially abundant proteins (red). Filtering was based on p< 0.05 and Log2 fold change > 1. Log2 fold change >1 = more abundant proteins, log2 fold change<1 less abundant proteins. Proteomics total variables =7577, RNA-seq total variables =27106. **(C, D)** Volcano plots of RIL175 WT vs TPC2 KO proteomic **(C)** and RNA-seq **(D)** data displaying all differentially abundant proteins (red). Filtering was based on p< 0.05 and Log2 fold change > 1. Log2 fold change >1 = more abundant proteins, log2 fold change<1 less abundant proteins. Proteomics total variables =1960, RNA-seq total variables =24321. **(E-H)** Principal component analysis showing sample-wise grouping according to experimental conditions from proteome analysis **(E, F)** and RNA-seq **(G, H). (I, J)** Heat map demonstrating label-free quantification intensities for all genes in all three RNA-seq experimental setups with dendrogram-based grouping according to experimental conditions.

Volcano plots of Hep3B proteome (**Figure 5A**) and transcriptome (**Figure 5B**) revealed that TPC2 KO cells displayed a broader and more significant pattern of gene and protein deregulation compared to TRPML1 KO (**Supplementary Figures S5A-D**). In contrast, in RIL175 cells, TRPML1 KO showed slightly more prominent deregulation in the proteomic and transcriptomic data (**Figures 5C, D**), though to a lesser extent than TPC2 KO in Hep3B.

In line, PCA of the proteomes clearly separated TPC2 KO from WT samples along PC1 in Hep3B (**Figure 5E**) and along PC2 in RIL175 (**Figure 5F**), explaining the greatest proportion of variance. Transcriptomic PCA further confirmed this marked shift in expression profiles (**Figures 5G, H**). In contrast, TRPML1 KO samples clustered closely with WT in both cell lines, indicating only minor molecular alterations (**Figures 5E, G**). Heatmap analysis revealed significant downregulation of genes critical for HCC proliferation and tumour progression in TPC2 KO cells, whereas TRPML1 KO cells maintained gene expression patterns like WT (**Figures 5I, J**, **S5E, F**). Many genes including Gramd1a, Sephin1, NPTX1, and Steap3 in RIL175 cells, and TM4SF1, PDGFRA, and AOX1 in Hep3B cells are linked to stemness, Ras/MAPK signalling, or poor prognosis in HCC (56–61). Moreover, genes implicated in immune evasion such as FJX1 and Aldoc were also downregulated specifically in TPC2 KO cells, supporting a dual role for TPC2 in tumour proliferation and immune escape (62, 63). Most importantly, in Hep3B cells proteome analysis revealed MHC-I to be significantly upregulated in TPC2 KO cells, whereas no significance was observed in ML1 KO cells. No significant changes in HLA-A or HLA-B expression could be observed through RNA-sequencing. In RIL175 cells MHC-I was not detected in proteome analysis but significantly upregulated in TPC2 KO cells RNA sequencing dataset.

In melanoma cells no clustering differences between TPC2 KO and TRPML1 KO cells was observed (**Supplementary Figures S5G-J**). Previous findings highlighted that both single KOs clustered closely together, whilst being separated from the WT and heatmaps did not show differences between TPC2 KO and TRPML1 KO cells (64). Here, with our murine proteomic melanoma data set we confirmed that TPC2 or TRPML1 KO does not appear to significantly change PCA clustering or alterations in protein expression pattern compared to WT cells (**Supplementary Figures S5G-J**).

**Figure 6:**
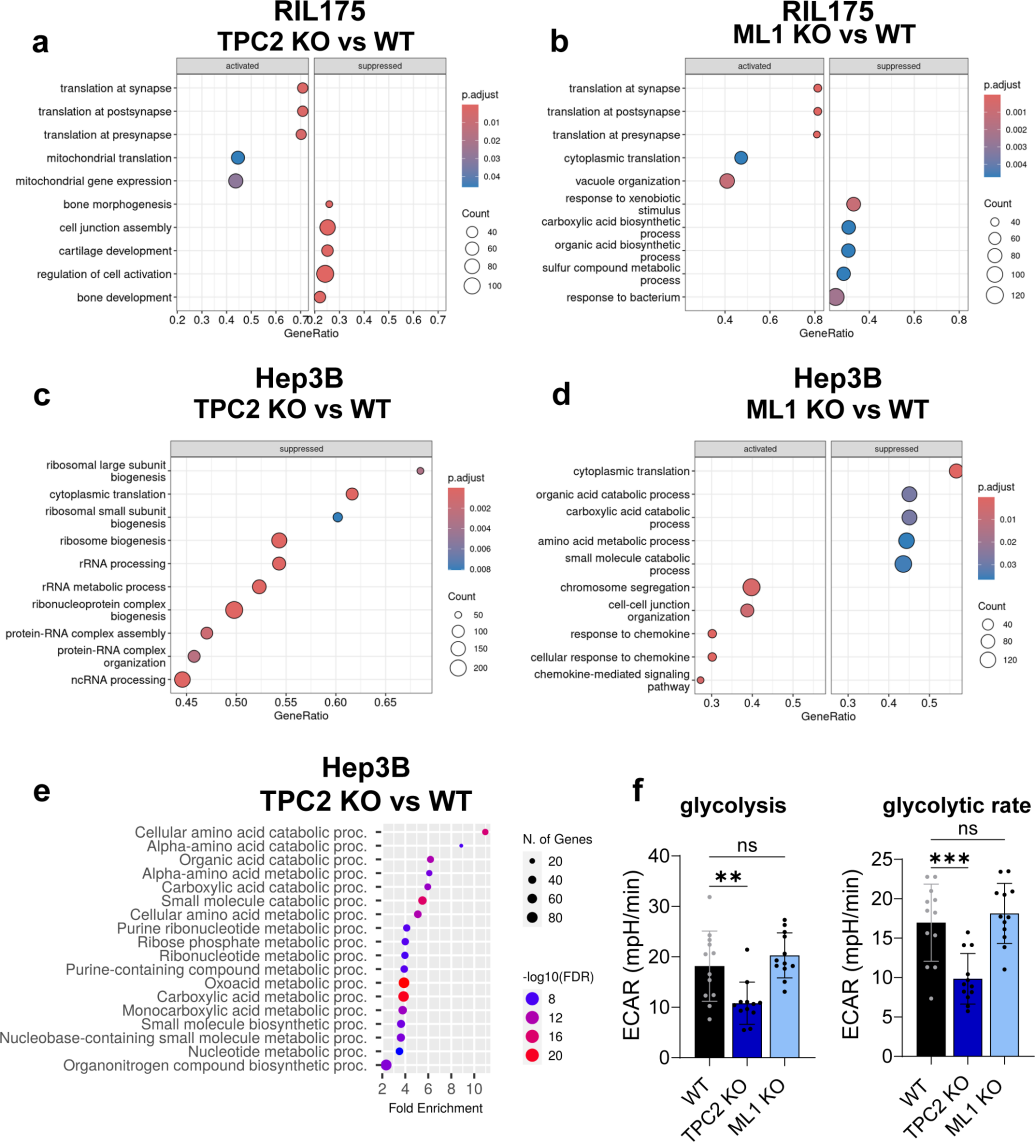
Gene set enrichment analysis of proteomics and RNA sequencing data. **(A, B)** GSEA of Hep3B RNA Sequencing data, shown are top 10 altered biological process (BP) pathways of TPC2 KO vs. WT **(A)** and ML1 KO vs. WT **(B)**. Suppressed = pathways with less abundant genes, activated = pathways with more abundant genes. **(C, D)** GSEA of RIL175 RNA Sequencing data, shown are top 10 altered biological process pathways of TPC2 KO vs. WT **(A)** and ML1 KO vs. WT **(B)**. Suppressed = pathways with less abundant genes, activated = pathways with more abundant genes. **(E)** GSEA of Hep3B proteome data TPC2 vs. WT top 20 BP pathways. Graphic created via ShinyGO 0.76. **(F)** Glycolytic stress test using Seahorse XFe96 Analyzer of Hep3B WT, TPC2 KO and ML1 KO cells reveals downregulated glycolysis and glycolytic rate in TPC2 KO cells (n=3). Statistical significance was assessed by One-way ANOVA Dunnett’s multiple comparisons test ** p< 0.01, *** p< 0.001, ns, not significant.

Gene Ontology (GO) analysis of our RNA-seq data identified the 10 most altered biological processes (BP). In RIL175 cells, TPC2 KO specifically downregulated pathways associated with cell activation and junction assembly (**Figure 6A**), both implicated in HCC and melanoma progression (8, 12, 13). Additionally, we newly identified that in RIL175 cells TPC2 and TRPML1 KOs lead to impaired translation processes (**Figures 6A, B**). In Hep3B, TPC2 KO also led to downregulation of RNA processing and translation pathways (**Figure 6C**), whereas TRPML1 KO reduced catabolic processes and upregulated chemokine-related responses (**Figure 6D**).

Gene Set Enrichment Analysis (GSEA) of proteome further confirmed this pattern (**Figure 6E**). In TRPML1 KO cells, altered pathways involved protein synthesis and degradation, mitochondrial gene expression, extracellular matrix organisation (**Supplementary Figure S6A**), which is in line with the literature (29, 30). TRPML1 KO cells also displayed metabolic shifts towards increased glycolysis, amino acid, and nucleotide metabolism, all indicating a proliferative state (**Supplementary Figures S6A, B**). Whereas, TPC2 KO resulted in a coherent downregulation of metabolic processes, including biosynthesis of amino acids, nucleotides, and organic acids (**Figure 6E**). Notably, key intermediates of glycolysis and the TCA cycle were affected (**Supplementary Figure S6C**), suggesting reduced energy production and impaired metabolic plasticity. Kyoto Encyclopaedia of Genes and Genomes (KEGG) pathway analysis supported this, revealing downregulation of glucose transporter genes (GLUT1/2), indicating reduced glucose uptake (**Supplementary Figure S6C**). In line, TPC2 KO cells exhibited significantly reduced glycolysis and glycolytic capacity compared to WT and TRPML1 KO cells (**Figures 6F**, **S6D**). This adds up to previous findings (13) describing the distinct role of TPC2 in regulating glycolytic metabolism in HCC cells, which is not shared by TRPML1.

Together, these findings establish that loss of TPC2, but not TRPML1, leads to extensive transcriptomic and proteomic alterations. TPC2 KO drives suppression of metabolic and proliferative signalling, particularly glycolysis and MAPK-associated processes. This dual shift in translational processes and metabolism underpins the profound phenotypic impact observed in TPC2-deficient tumours.

### 3.6 Translational suppression of MAPK components in TPC2 KO cells reduces tumour proliferation

**Figure 7:**
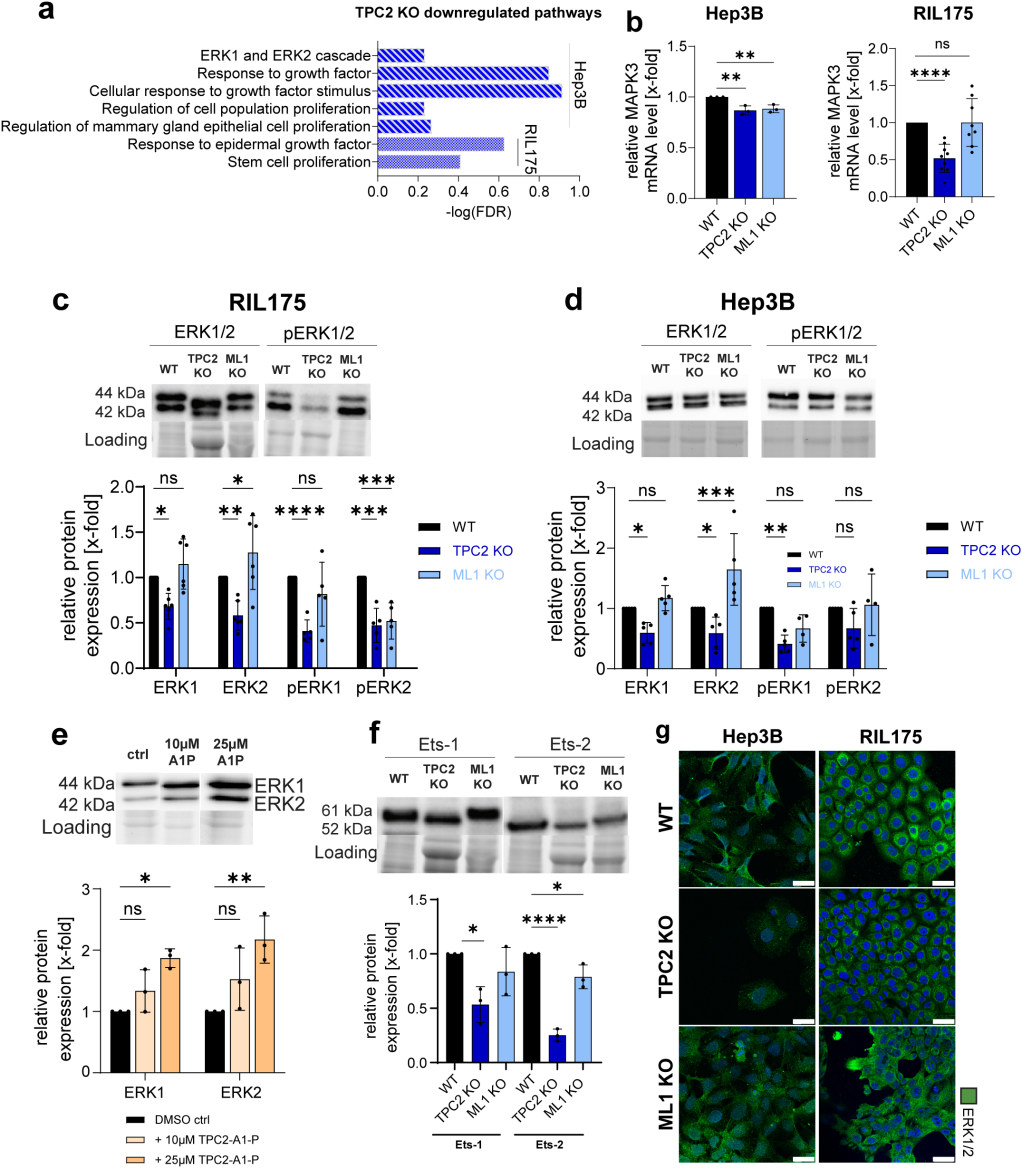
TPC2 KO impairs ERK1/2 signalling. **(A)** GSEA of Hep3B and RIL175 TPC2 KO vs WT show altered BP pathways upon KO of TPC2. **(B)** RT-qPCR of MAPK3 (ERK1). Actin served as housekeeping gene. (Hep3B n=3, RIL175 n=8) **(C, D)** Relative protein levels of ERK1/2 and pERK1/2 (n=5) **(E)** Relative protein levels of ERK1/2 and pERK1/2 in RIL175 WT cells after 24h stimulation with TPC2-A1-P (n=3). **(F)** Relative protein levels of Ets-1 and Ets-2 (n=3). **(G)** Confocal images of ERK1/2. Nuclei stained with Hoechst (blue) and quantification. Scale bars 25µm. Representative images shown (all n=3). Statistical significance was assessed by One-way ANOVA Dunnett’s multiple comparisons test * p< 0.05, ** p< 0.01, *** p< 0.001, **** p<0.0001, ns, not significant. Images/Blots are representative.

Functional GSEA revealed a marked downregulation of proliferation-associated biological processes in TPC2 KO cells (**Figure 7A**), consistent with the reduced tumour growth observed *in vitro* and *in vivo* (**Figure 2**). Additionally, complementary KEGG analysis of the Hep3B proteomic dataset identified significant suppression of Receptor Tyrosine Kinase (RTK) signalling, pinpointing the MAPK pathway as a primary axis affected by TPC2 loss, with MAPK3 (ERK1) emerging as a key downregulated target (**Figure 5A, C**, **Supplementary Figure S5D**). Notably, MAPK pathway activity has been shown to negatively regulate MHC-I surface expression on tumour cells, hence influencing immune recognition by cytotoxic T cells (65–67). As we observed a significant reduction in MHC-I expression in TPC2 KO tumours *ex vivo* (**Figures 3A, B**) and TPC2 KO cell *in vitro* (**Figures 2A, C**), we hypothesize that a reduction of MHC-I might be linked to downregulated ERK1/2 expression.

In fact, MAPK3 mRNA levels (**Figure 7B**) were significantly downregulated in TPC2 KO cell lines. In line with proteomic data (**Figures 6A, C**), total ERK1/2 protein levels were significantly decreased in TPC2 KO HCC cell lines (**Figures 7C-F, G**, **S7A**), while melanoma cell lines and TRPML1 KO cells showed no such reduction (**Supplementary Figures S7B, C**). We also found phosphorylated, i.e. activated, ERK1/2 levels reduced in all TPC2 KO cell lines (**Figures 7C, D**). However, the relative amount of phosphorylation remained stable in HCC (**Supplementary Figure S7D**). This indicates that the reduced signalling is primarily driven by a loss in total ERK1/2 protein abundance, rather than impaired phosphorylation. In line, direct activation of TPC2 resulted in elevated ERK1/2 protein expression (**Figure 7E**). Lastly, expression of ERK downstream effectors Ets-1 and Ets-2, known for their cancer promoting roles, was also reduced (68, 69) (**Figure 7F**). These findings support our earlier transcriptomic and proteomic data (**Figures 6A, C**), suggesting that TPC2 KO leads to decreased ERK1/2 levels through suppressed transcriptional and translational activity. These reductions were further confirmed via confocal microscopy (**Figures 7G**, **S7A, S7F, G**).

Since RTKs also modulate MAPK/JNK signalling, we further examined JNK protein expression. While total JNK remained unchanged, phosphorylated JNK was significantly reduced in HCC TPC2 KO cells (**Supplementary Figures S7H, J**). Additionally, the downstream effector c-Jun and its phosphorylated form were consistently reduced in HCC TPC2 KO cells (**Supplementary Figures S7I, K**), further supporting suppression of MAPK/JNK signalling upon TPC2 ablation. In contrast, in TRPML1 KO total and phosphorylated JNK levels remained largely unaffected across most cell lines (**Supplementary Figures S7H, J**) and did not exhibit consistent alterations in downstream JNK pathway components (**Supplementary Figures S7I, K**). These findings suggest that TRPML1 loss does not result in a coherent phenotype with respect to JNK signalling, further highlighting the specific regulatory role of TPC2 in modulating MAPK/JNK pathway activity.

These results propose that the strong antiproliferative effects observed in HCC TPC2 KO cells arise from impaired protein translation, which subsequently leads to disruption of MAPK/ERK and MAPK/JNK signalling pathways. The absence of comparable effects in melanoma cells suggests a cancer-type-specific function of TPC2. Importantly, the downregulation of MAPK signalling, particularly reduced ERK1/2 expression, correlates with increased MHC-I surface levels in TPC2 KO cells. This supports a model in which impaired ERK signalling leads to MAPK-driven repression of antigen presentation pathways, thereby enhancing MHC-I expression and tumour immunogenicity.

## 4 Discussion

Previously, members of the TRPML and TPC families have been implicated in regulating cytokine secretion in immune cells, e.g. Plesch et al. have shown that TRPML2 mediates CCL2 release in macrophages (70) and histamine secretion in mast cells is TPC1 dependent (19). Still, a direct role for these ion channels in modulating anti-tumour immunity or cytokine responses in cancer cells has not been previously described. Our study identifies TPC2 as a central regulator of tumour progression, exerting dual effects on cancer cell proliferation and immune evasion in HCC. In contrast, a KO of lysosomal calcium channel TRPML1 did not affect tumour progression *in vivo*, underscoring the specificity of TPC2 in driving this phenotype. TPC2 KO led to a robust reduction in tumour growth, particularly in HCC (**Figures 2J-L**). A comparative analysis of melanoma and HCC cell lines and tumour models revealed that the MAPK-dependent effects of TPC2 depletion (**Figure 7**), affecting both proliferation and immunogenicity, were specific to HCC, underscoring a tumour-type-dependent role for TPC2 in cancer progression.

*Ex vivo* analysis of HCC tumour sections revealed massive necrosis and increased infiltration of CD8^+^ T cells in TPC2 KO tumours (**Figure 3A**), suggesting enhanced cytotoxic immune responses. In line with this, co-culture experiments showed elevated IFN-γ secretion and increased CD8 expression on T cells exposed to TPC2-deficient tumour cells (**Figure 3I**). In HCC tumour microenvironment (TME), CD8^+^ T cells frequently undergo exhaustion or dysfunction, losing their cytotoxic potential and reducing pro-inflammatory cytokine production (71, 72). This dysfunction of CD8^+^ T cells is associated with elevated regulatory T cell levels and poor prognosis in hepatocellular carcinoma patients (73), posing a major challenge for effective therapy. Here, we show that TPC2 loss can reverse this exhaustion, effectively priming CD8^+^ T cells for anti-tumour activity. We further propose that the enhanced T cell response is supported by increased MHC-I and decreased PD-L1 expression on TPC2-deficient HCC cells.

MHC-I is essential for effective antigen presentation to CD8^+^ T cells (74), and the expression of checkpoint inhibitor PD-L1 on the cancer cell surface has been shown to contribute to immunotherapy resistance (75). Here we show that namely TPC2 KO increases MHC-I surface expression and reduces PD-L1 levels on tumour cells, most prominently in RIL175 cells (**Figure 4**), which also exhibited the strongest tumour suppression. IHC confirmed these trends, reinforcing the role of TPC2 in immune evasion (**Figure 3A**).

Our findings are consistent with those of He et al., who reported that tetrandrine, a TPC2 antagonist, increased MHC-I expression and IFN-γ secretion in melanoma, thereby enhancing responsiveness to PD-1 blockade (21). However, due to tetrandrine’s broad pharmacological profile and potential off-target effects (76), we are the first to demonstrate that modulation of MHC-I and PD-L1 is specifically mediated by TPC2, using both stable KO, transient KD models and more selective pharmacological tools (SG-094 and TPC2-A1-P, **Figures 4C-H**). While the study of He et al. focused on melanoma, our direct comparison reveals that TPC2 KO in melanoma leads to less consistent changes in MHC-I and PD-L1, paralleling the weaker *in vivo* phenotype. Mechanistically, previous studies have already linked TPC2 to melanoma proliferation via the Rab7/MITF axis (12) and He et al. linked MHC-I expression to TPC2-dependant autophagy (21). In contrast, our data newly establish a mechanistic role for TPC2 in HCC, where its loss significantly suppresses MAPK signalling, reducing total ERK levels and its downstream targets in HCC (**Figure 7**, **S7**). Conversely, direct activation of TPC2 with agonist TPC2-A1-P resulted in elevated total ERK1/2 protein levels (**Figure 7E**), indicating that changes in ERK levels are TPC2-dependant. Since MAPK inhibition is known to promote antigen presentation and enhance T cell cytotoxicity (65, 66), we propose a TPC2/MAPK/MHC-I axis as a key regulator of tumour immunity in HCC. This pathway appears to be cancer-type specific, as MAPK impairment was not observed in melanoma models (12, 77), underscoring differential regulatory mechanisms by TPC2 across tumour types.

Beyond immune modulation, we also propose cancer proliferation in HCC to be regulated via TPC2/MAPK axis, as the Ras/Raf/MAPK pathway is a well-established driver of tumour growth and survival and is hyperactivated in HCC (78, 79). Moreover, the MAPK pathway is also linked to metabolic reprogramming and the Warburg effect (80). Our omics and Seahorse data revealed a shift in glycolytic metabolism (**Figure 6**), suggesting that TPC2 affects metabolism through MAPK regulation. Müller et al. observed a similar shift in liver cancer cells upon KO but did not link it to MAPK signalling (13). Furthermore, unbiased proteomic and transcriptomic profiling revealed that TPC2 deletion disrupts translational processes in HCC. KO cells exhibited higher lysosomal pH, enlarged lysosomes, and delayed acidification (**Figure 1**), pointing to lysosomal dysfunction. As lysosomes regulate protein turnover and cellular homeostasis (81), their dysfunction likely contributes to impaired translation (82, 83). ERK1/2 mRNA and protein levels were significantly downregulated (**Figure 7**), not due to impaired phosphorylation but reduced synthesis, linking TPC2 loss to a global shift in protein production and MAPK suppression.

Due to the central role of MAPK hyperactivation in HCC, pharmacological efforts have focused on kinase inhibitors such as sorafenib and lenvatinib. However, these therapies offer limited efficacy due to high rates of chemoresistance and poor overall survival (84). In parallel, immunotherapy targeting PD-1/PD-L1 has significantly improved outcomes in a subset of patients while reducing adverse effects, yet many patients either fail to respond or develop resistance during treatment (85, 86). Our findings identify TPC2 as a dual-action therapeutic target: its inhibition not only disrupts Ras/Raf/MAPK-driven tumour proliferation but also enhances tumour immunogenicity by increasing MHC-I and reducing PD-L1 expression. Notably, previous studies have shown that TPC2 KO can overcome chemoresistance (44), underscoring its clinical relevance in treatment-refractory HCC. Supporting this concept, our combination treatment experiments revealed that co-administration of the TPC2 inhibitor SG094 and the PD-1 checkpoint inhibitor Nivolumab significantly elevated IFN-γ secretion in co-cultured CD8^+^ T cells (**Figure 4I**). These results raise the compelling possibility that TPC2 inhibition could serve as an effective adjuvant to immune checkpoint inhibitor therapy. Together, this dual mechanism of action positions TPC2 as a promising target for combination strategies aimed at expanding immunotherapy efficacy, particularly in early and intermediate-stage HCC.

Taken together, our study provides the first mechanistic link of TPC2 with immune evasion and cancer proliferation via MAPK/ERK signalling in HCC. We propose a loss of TPC2 function to be responsible for impaired translation of ERK1/2 and therefore impaired MAPK/ERK signalling, subsequently leading to elevated MHC-1 levels and reduced tumour growth. Thus, we identify TPC2 as a central regulator of tumour progression and immune evasion, offering a promising therapeutic target to simultaneously impair cancer proliferation, overcome chemoresistance, and boost anti-tumour immunity particularly in HCC.

This article contains **Supplementary Material**.

## Data Availability

The datasets presented in this study can be found in online repositories. The names of the repository/repositories and accession number(s) can be found below (87): http://www.proteomexchange.org/, reviewer_pxd062988@ebi.ac.uk, https://www.ncbi.nlm.nih.gov/, PRJNA1255649.

## References

[1] Torre LA Bray F Siegel RL Ferlay J Lortet‐Tieulent J Jemal A. Global cancer statistics, 2012. Cancer J Clin. (2015) 65:87–108. doi: 10.3322/caac.21262, PMID: 25651787

[2] European Association For The Study Of The Liver, European Organisation For Research And Treatment Of Cancer. EASL-EORTC clinical practice guidelines: management of hepatocellular carcinoma. J Hepatol. (2012) 56:908–43. doi: 10.1016/j.jhep.2011.12.001, PMID: 22424438

[3] Wong VW-S Chitturi S Wong GL-H Yu J Chan HL-Y Farrell GC. Pathogenesis and novel treatment options for non-alcoholic steatohepatitis. Lancet Gastroenterol Hepatol. (2016) 1:56–67. doi: 10.1016/S2468-1253(16)30011-5, PMID: 28404113

[4] Zucman-Rossi J Villanueva A Nault J-C Llovet JM. Genetic landscape and biomarkers of hepatocellular carcinoma. Gastroenterology. (2015) 149:1226–1239.e4. doi: 10.1053/j.gastro.2015.05.061, PMID: 26099527

[5] Kinsey E Lee HM. Management of hepatocellular carcinoma in 2024: the multidisciplinary paradigm in an evolving treatment landscape. Cancers. (2024) 16. doi: 10.3390/cancers16030666, PMID: 38339417 PMC10854554

[6] MaChado ER Annunziata I van de Vlekkert D Grosveld GC d'Azzo A. Lysosomes and cancer progression: A Malignant liaison. Front Cell Dev Biol. (2021) 9:642494. doi: 10.3389/fcell.2021.642494, PMID: 33718382 PMC7952443

[7] Deutsch R Kudrina V Freichel M Grimm C. Two-pore channel regulators - Who is in control? Front Physiol. (2024) 15:1534071. doi: 10.3389/fphys.2024.1534071, PMID: 39867224 PMC11757267

[8] Nguyen ONP Grimm C Schneider LS Chao Y-K Atzberger C Bartel K. Two-pore channel function is crucial for the migration of invasive cancer cells. Cancer Res. (2017) 77:1427–38. doi: 10.1158/0008-5472.CAN-16-0852, PMID: 28108508

[9] Favia A Desideri M Gambara G D’Alessio A Ruas M Esposito B. VEGF-induced neoangiogenesis is mediated by NAADP and two-pore channel-2-dependent Ca2+ signaling. Proc Natl Acad Sci United States America. (2014) 111:E4706–15. doi: 10.1073/pnas.1406029111, PMID: 25331892 PMC4226099

[10] Favia A Pafumi I Desideri M Padula F Montesano C Passeri D. NAADP-dependent ca(2+) signaling controls melanoma progression, metastatic dissemination and neoangiogenesis. Sci Rep. (2016) 6:18925. doi: 10.1038/srep18925, PMID: 26733361 PMC4702115

[11] Sun W Yue J. TPC2 mediates autophagy progression and extracellular vesicle secretion in cancer cells. Exp Cell Res. (2018) 370:478–89. doi: 10.1016/j.yexcr.2018.07.013, PMID: 29990474

[12] Abrahamian C Tang R Deutsch R Ouologuem L Weiden E-M Kudrina V. Rab7a is an enhancer of TPC2 activity regulating melanoma progression through modulation of the GSK3β/β-Catenin/MITF-axis. Nat Commun. (2024) 15:10008. doi: 10.1038/s41467-024-54324-9, PMID: 39562548 PMC11576762

[13] Müller M Gerndt S Chao Y-K Zisis T Nguyen ONP Gerwien A. Gene editing and synthetically accessible inhibitors reveal role for TPC2 in HCC cell proliferation and tumor growth. Cell Chem Biol. (2021) 28:1119–1131.e27. doi: 10.1016/j.chembiol.2021.01.023, PMID: 33626324

[14] Ouologuem L Bartel K. Endolysosomal transient receptor potential mucolipins and two-pore channels: implications for cancer immunity. Front Immunol. (2024) 15:1389194. doi: 10.3389/fimmu.2024.1389194, PMID: 38840905 PMC11150529

[15] Goretzko J Heitzig N Thomas K Krogsaeter EK Naß J Matos AL. Leukocyte adhesion is governed by endolysosomal two pore channel 2 (TPC2). Cell Rep. (2021). 10.1016/j.celrep.2023.113501 38039128

[16] Goretzko J Pauels I Heitzig N Thomas K Kardell M Naß J. P-selectin-dependent leukocyte adhesion is governed by endolysosomal two-pore channel 2. Cell Rep. (2023) 42:113501. doi: 10.1016/j.celrep.2023.113501, PMID: 38039128

[17] Davis LC Morgan AJ Galione A. NAADP-regulated two-pore channels drive phagocytosis through endo-lysosomal Ca2+ nanodomains, calcineurin and dynamin. EMBO J. (2020) 39:e104058. doi: 10.15252/embj.2019104058, PMID: 32510172 PMC7360967

[18] Davis LC Morgan AJ Chen J-L Snead CM Bloor-Young D Shenderov E. NAADP activates two-pore channels on T cell cytolytic granules to stimulate exocytosis and killing. Curr Biol: CB. (2012) 22:2331–7. doi: 10.1016/j.cub.2012.10.035, PMID: 23177477 PMC3525857

[19] Arlt E Fraticelli M Volodymyr V Nadolni W Breit A O’Neill TJ. TPC1 deficiency or blockade augments systemic anaphylaxis and mast cell activity. Proc Natl Acad Sci United States America. (2020) 117:18068–78. doi: 10.1073/pnas.1920122117, PMID: 32661165 PMC7395440

[20] Yamamoto K Venida A Yano J Biancur DE Kakiuchi M Gupta S. Autophagy promotes immune evasion of pancreatic cancer by degrading MHC-I. Nature. (2020) 581:100–5. doi: 10.1038/s41586-020-2229-5, PMID: 32376951 PMC7296553

[21] He L Liu Y Jiang J Wang D Li Y Zeng S. Tetrandrine augments melanoma cell immunogenicity via dual inhibition of autophagic flux and proteasomal activity enhancing MHC-I presentation. Acta Pharmacol Sin. (2025) 46 2056–72. doi: 10.1038/s41401-025-01507-9, PMID: 40016522 PMC12205077

[22] Contardi E Palmisano GL Tazzari PL Martelli AM Falà F Fabbi M. CTLA-4 is constitutively expressed on tumor cells and can trigger apoptosis upon ligand interaction. Int J Cancer. (2005) 117:538–50. doi: 10.1002/ijc.21155, PMID: 15912538

[23] Kashiwada T Takano R Ando F Kuroda S Miyabe Y Owada R. Lysosomal degradation of PD-L1 is associated with immune-related adverse events during anti-PD-L1 immunotherapy in NSCLC patients. Front Pharmacol. (2024) 15:1384733. doi: 10.3389/fphar.2024.1384733, PMID: 38799168 PMC11116720

[24] Lin X Kang K Chen P Zeng Z Li G Xiong W. Regulatory mechanisms of PD-1/PD-L1 in cancers. Mol Cancer. (2024) 23:108. doi: 10.1186/s12943-024-02023-w, PMID: 38762484 PMC11102195

[25] Schoenfeld AJ Hellmann MD. Acquired resistance to immune checkpoint inhibitors. Cancer Cell. (2020) 37:443–55. doi: 10.1016/j.ccell.2020.03.017, PMID: 32289269 PMC7182070

[26] Bai J Gao Z Li X Dong L Han W Nie J. Regulation of PD-1/PD-L1 pathway and resistance to PD-1/PD-L1 blockade. Oncotarget. (2017) 8:110693–707. doi: 10.18632/oncotarget.22690, PMID: 29299180 PMC5746415

[27] Yao H Lan J Li C Shi H Brosseau J-P Wang H. Inhibiting PD-L1 palmitoylation enhances T-cell immune responses against tumours. Nat Biomed Eng. (2019) 3:306–17. doi: 10.1038/s41551-019-0375-6, PMID: 30952982

[28] Orci LA Lacotte S Delaune V Slits F Oldani G Lazarevic V. Effects of the gut-liver axis on ischaemia-mediated hepatocellular carcinoma recurrence in the mouse liver. J Hepatol. (2018) 68:978–85. doi: 10.1016/j.jhep.2017.12.025, PMID: 29331341

[29] Siow WX Kabiri Y Tang R Chao Y-K Plesch E Eberhagen C. Lysosomal TRPML1 regulates mitochondrial function in hepatocellular carcinoma cells. J Cell Sci. (2022) 135. doi: 10.1242/jcs.259455, PMID: 35274126 PMC8977057

[30] Frey N Ouologuem L Blenninger J Siow W-X Thorn-Seshold J Stöckl J. Endolysosomal TRPML1 channel regulates cancer cell migration by altering intracellular trafficking of E-cadherin and β1-integrin. J Biol Chem. (2024) 300:105581. doi: 10.1016/j.jbc.2023.105581, PMID: 38141765 PMC10825694

[31] Bauer DE Canver MC Orkin SH. Generation of genomic deletions in mammalian cell lines via CRISPR/Cas9. J Visual Experiments: JoVE. (2015) 95:e52118. doi: 10.3791/52118, PMID: 25549070 PMC4279820

[32] Varghese F Bukhari AB Malhotra R De A. IHC Profiler: an open source plugin for the quantitative evaluation and automated scoring of immunohistochemistry images of human tissue samples. PloS One. (2014) 9:e96801. doi: 10.1371/journal.pone.0096801, PMID: 24802416 PMC4011881

[33] Chandra A Prasad S Alemanno F De Luca M Rizzo R Romano R. Fully automated computational approach for precisely measuring organelle acidification with optical pH sensors. ACS Appl Mater Interfac. (2022) 14:18133–49. doi: 10.1021/acsami.2c00389, PMID: 35404562 PMC9052195

[34] Fleige S Walf V Huch S Prgomet C Sehm J Pfaffl MW. Comparison of relative mRNA quantification models and the impact of RNA integrity in quantitative real-time RT-PCR. Biotechnol Lett. (2006) 28:1601–13. doi: 10.1007/s10529-006-9127-2, PMID: 16900335

[35] Demichev V Messner CB Vernardis SI Lilley KS Ralser M. DIA-NN: neural networks and interference correction enable deep proteome coverage in high throughput. Nat Methods. (2020) 17:41–4. doi: 10.1038/s41592-019-0638-x, PMID: 31768060 PMC6949130

[36] Ge SX Jung D Yao R. ShinyGO: a graphical gene-set enrichment tool for animals and plants. Bioinf (Oxford England). (2020) 36:2628–9. doi: 10.1093/bioinformatics/btz931, PMID: 31882993 PMC7178415

[37] Luo W Brouwer C. Pathview: an R/Bioconductor package for pathway-based data integration and visualization. Bioinf (Oxford England). (2013) 29:1830–1. doi: 10.1093/bioinformatics/btt285, PMID: 23740750 PMC3702256

[38] Ewels P Magnusson M Lundin S Käller M. MultiQC: summarize analysis results for multiple tools and samples in a single report. Bioinf (Oxford England). (2016) 32:3047–8. doi: 10.1093/bioinformatics/btw354, PMID: 27312411 PMC5039924

[39] Dobin A Davis CA Schlesinger F Drenkow J Zaleski C Jha S. STAR: ultrafast universal RNA-seq aligner. Bioinf (Oxford England). (2013) 29:15–21. doi: 10.1093/bioinformatics/bts635, PMID: 23104886 PMC3530905

[40] Dyer SC Austine-Orimoloye O Azov AG Barba M Barnes I Barrera-Enriquez VP. Ensembl 2025. Nucleic Acids Res. (2025) 53:D948–57. doi: 10.1093/nar/gkae1071, PMID: 39656687 PMC11701638

[41] Liao Y Smyth GK Shi W. featureCounts: an efficient general purpose program for assigning sequence reads to genomic features. Bioinf (Oxford England). (2014) 30:923–30. doi: 10.1093/bioinformatics/btt656, PMID: 24227677

[42] Love MI Huber W Anders S. Moderated estimation of fold change and dispersion for RNA-seq data with DESeq2. Genome Biol. (2014) 15:550. doi: 10.1186/s13059-014-0550-8, PMID: 25516281 PMC4302049

[43] Wu T Hu E Xu S Chen M Guo P Dai Z. clusterProfiler 4.0: A universal enrichment tool for interpreting omics data. Innovation (Cambridge (Mass.)). (2021) 2:100141. doi: 10.1016/j.xinn.2021.100141, PMID: 34557778 PMC8454663

[44] Geisslinger F Müller M Chao Y-K Grimm C Vollmar AM Bartel K. Targeting TPC2 sensitizes acute lymphoblastic leukemia cells to chemotherapeutics by impairing lysosomal function. Cell Death Dis. (2022) 13:668. doi: 10.1038/s41419-022-05105-z, PMID: 35915060 PMC9343397

[45] Lu Y Hao B-X Graeff RM Wong CWM Wu W-T Yue J. Two pore channel 2 (TPC2) inhibits autophagosomal-lysosomal fusion by alkalinizing lysosomal pH. J Biol Chem. (2013) 288:24247–63. doi: 10.1074/jbc.M113.484253, PMID: 23836916 PMC3745369

[46] Ohkuma S Poole B. Fluorescence probe measurement of the intralysosomal pH in living cells and the perturbation of pH by various agents. Proc Natl Acad Sci United States America. (1978) 75:3327–31. doi: 10.1073/pnas.75.7.3327, PMID: 28524 PMC392768

[47] ThermoFisher Scientific. LysoSensor and LysoTracker Probes – Product Information and Technical Resources. ThermoFisher Sci. (2021).

[48] Johnson DE Ostrowski P Jaumouillé V Grinstein S. The position of lysosomes within the cell determines their luminal pH. J Cell Biol. (2016) 212:677–92. doi: 10.1083/jcb.201507112, PMID: 26975849 PMC4792074

[49] Verma A Stellacci F. Effect of surface properties on nanoparticle-cell interactions. Small (Weinheim an der Bergstrasse Germany). (2010) 6:12–21. doi: 10.1002/smll.200901158, PMID: 19844908

[50] Iuele H Forciniti S Onesto V Colella F Siciliano AC Chandra A. Facile one pot synthesis of hybrid core-shell silica-based sensors for live imaging of dissolved oxygen and hypoxia mapping in 3D cell models. ACS Appl Mater Interfac. (2024) 41:55071–85. doi: 10.1021/acsami.4c08306, PMID: 39205375 PMC13425555

[51] Rizzo R Onesto V Forciniti S Chandra A Prasad S Iuele H. A pH-sensor scaffold for mapping spatiotemporal gradients in three-dimensional *in vitro* tumour models. Biosensors Bioelectron. (2022) 212:114401. doi: 10.1016/j.bios.2022.114401, PMID: 35617754

[52] Rizzo R Onesto V Morello G Iuele H Scalera F Forciniti S. pH-sensing hybrid hydrogels for non-invasive metabolism monitoring in tumor spheroids. Mater Today Bio. (2023) 20:100655. doi: 10.1016/j.mtbio.2023.100655, PMID: 37234366 PMC10205545

[53] Han J Burgess K. Fluorescent indicators for intracellular pH. Chem Rev. (2010) 110:2709–28. doi: 10.1021/cr900249z, PMID: 19831417

[54] Jaqaman K Loerke D Mettlen M Kuwata H Grinstein S Schmid SL. Robust single-particle tracking in live-cell time-lapse sequences. Nat Methods. (2008) 5:695–702. doi: 10.1038/nmeth.1237, PMID: 18641657 PMC2747604

[55] Vackova J Polakova I Johari SD Smahel M. CD80 expression on tumor cells alters tumor microenvironment and efficacy of cancer immunotherapy by CTLA-4 blockade. Cancers. (2021) 13. doi: 10.3390/cancers13081935, PMID: 33923750 PMC8072777

[56] Zhao Y Yu Y Zhao W You S Feng M Xie C. As a downstream target of the AKT pathway, NPTX1 inhibits proliferation and promotes apoptosis in hepatocellular carcinoma. Biosci Rep. (2019) 39. doi: 10.1042/BSR20181662, PMID: 31113871 PMC6549097

[57] Zhong H Wang Z Wei X Liu Y Huang X Mo X. Prognostic and immunological role of SERPINH1 in pan-cancer. Front Genet. (2022) 13:900495. doi: 10.3389/fgene.2022.900495, PMID: 36105106 PMC9465257

[58] Wang L-L Luo J He Z-H Liu Y-Q Li H-G Xie D. STEAP3 promotes cancer cell proliferation by facilitating nuclear trafficking of EGFR to enhance RAC1-ERK-STAT3 signaling in hepatocellular carcinoma. Cell Death Dis. (2021) 12:1052. doi: 10.1038/s41419-021-04329-9, PMID: 34741044 PMC8571373

[59] Fu B Meng W Zhao H Zhang B Tang H Zou Y. GRAM domain-containing protein 1A (GRAMD1A) promotes the expansion of hepatocellular carcinoma stem cell and hepatocellular carcinoma growth through STAT5. Sci Rep. (2016) 6:31963. doi: 10.1038/srep31963, PMID: 27585821 PMC5009375

[60] Guo J Chen L Dai B Sui C Dong Z Chen K. TM4SF1 overexpression in tumor-associated endothelial cells promotes microvascular invasion in hepatocellular carcinoma. Front Oncol. (2025) 15:1526177. doi: 10.3389/fonc.2025.1526177, PMID: 40123905 PMC11925789

[61] Liu Y Fu S Zhang Z Wang S Cheng X Li Z. GRAMD1A is a biomarker of kidney renal clear cell carcinoma and is associated with immune infiltration in the tumour microenvironment. Dis Markers. (2022) 2022:5939021. doi: 10.1155/2022/5939021, PMID: 35860689 PMC9293538

[62] Chen L Zeng Y Ren B Wang X Zhao F Du J. ALDOC regulated the biological function and immune infiltration of gastric cancer cells. Int J Biochem Cell Biol. (2023) 158:106407. doi: 10.1016/j.biocel.2023.106407, PMID: 36997056

[63] Huang M Guo T Meng Y Zhou R Xiong M Ding J. Comprehensive analysis of the prognosis and immune effect of the oncogenic protein Four Jointed Box 1. Front Oncol. (2023) 13:1170482. doi: 10.3389/fonc.2023.1170482, PMID: 37324001 PMC10266275

[64] Abrahamian C Ouologuem L Tang R Fröhlich T Bartel K Grimm C. TPC2: from blond hair to melanoma? Cancers. (2024) 16:4065. doi: 10.3390/cancers16234065, PMID: 39682251 PMC11640397

[65] Loi S Dushyanthen S Beavis PA Salgado R Denkert C Savas P. RAS/MAPK activation is associated with reduced tumor-infiltrating lymphocytes in triple-negative breast cancer: therapeutic cooperation between MEK and PD-1/PD-L1 immune checkpoint inhibitors. Clin Cancer Res. (2016) 22:1499–509. doi: 10.1158/1078-0432.CCR-15-1125, PMID: 26515496 PMC4794351

[66] Brea EJ Oh CY Manchado E Budhu S Gejman RS Mo G. Kinase regulation of human MHC class I molecule expression on cancer cells. Cancer Immunol Res. (2016) 4:936–47. doi: 10.1158/2326-6066.CIR-16-0177, PMID: 27680026 PMC5110210

[67] Franklin DA James JL Axelrod ML Balko JM. MEK inhibition activates STAT signaling to increase breast cancer immunogenicity via MHC-I expression. Cancer Drug Resist (Alhambra Calif.). (2020) 3:603–12. doi: 10.20517/cdr.2019.109, PMID: 33062958 PMC7556720

[68] Liu X Zhang C Zhang Z Zhang Z Ji W Cao S. E26 transformation-specific transcription factor ETS2 as an oncogene promotes the progression of hypopharyngeal cancer. Cancer Biother Radiopharmaceut. (2017) 32:327–34. doi: 10.1089/cbr.2017.2296, PMID: 29111780

[69] Wang H Chu F Li Z Bi Q Li L Zhua Y. MTBP enhances the activation of transcription factor ETS-1 and promotes the proliferation of hepatocellular carcinoma cells. Front Oncol. (2022) 12:985082. doi: 10.3389/fonc.2022.985082, PMID: 36106099 PMC9464980

[70] Plesch E. Selective agonist of TRPML2 reveals direct role in chemokine release from innate immune cells. eLife. (2018) 7. doi: 10.7554/eLife.39720, PMID: 30479274 PMC6257821

[71] Shirabe K. Tumor-infiltrating lymphocytes and hepatocellular carcinoma: pathology and clinical management. Int J Clin Oncol. (2010) 15:552–8. doi: 10.1007/s10147-010-0131-0, PMID: 20963618

[72] Gao Q. Intratumoral balance of regulatory and cytotoxic T cells is associated with prognosis of hepatocellular carcinoma after resection. J Clin Oncol. (2007) 25:2586–93. doi: 10.1200/JCO.2006.09.4565, PMID: 17577038

[73] Fu J. Increased regulatory T cells correlate with CD8 T-cell impairment and poor survival in hepatocellular carcinoma patients. Gastroenterology. (2007) 132:2328–39. doi: 10.1053/j.gastro.2007.03.102, PMID: 17570208

[74] Sari G Rock KL. Tumor immune evasion through loss of MHC class-I antigen presentation. Curr Opin Immunol. (2023) 83:102329. doi: 10.1016/j.coi.2023.102329, PMID: 37130455 PMC10524158

[75] Li Q Han J Yang Y Chen Y. PD-1/PD-L1 checkpoint inhibitors in advanced hepatocellular carcinoma immunotherapy. Front Immunol. (2022) 13:1070961. doi: 10.3389/fimmu.2022.1070961, PMID: 36601120 PMC9806143

[76] Song J. Therapeutic effects of tetrandrine in inflammatory diseases: a comprehensive review. Inflammopharmacology. (2024) 32:1743–57. doi: 10.1007/s10787-024-01452-9, PMID: 38568399

[77] Netcharoensirisuk P. Flavonoids increase melanin production and reduce proliferation, migration and invasion of melanoma cells by blocking endolysosomal/melanosomal TPC2. Sci Rep. (2021) 11:8515. doi: 10.1038/s41598-021-88196-6, PMID: 33875769 PMC8055690

[78] Sun Y. Signaling pathway of MAPK/ERK in cell proliferation, differentiation, migration, senescence and apoptosis. J Receptor Signal Transduct Res. (2015) 35:600–4. doi: 10.3109/10799893.2015.1030412, PMID: 26096166

[79] Hoffmann K. Correlation of gene expression of ATP-binding cassette protein and tyrosine kinase signaling pathway in patients with hepatocellular carcinoma. Anticancer Res. (2011) 31:3883–90., PMID: 22110214

[80] Papa S Choy PM Bubici C. The ERK and JNK pathways in the regulation of metabolic reprogramming. Oncogene. (2019) 38:2223–40. doi: 10.1038/s41388-018-0582-8, PMID: 30487597 PMC6398583

[81] Ballabio A Bonifacino JS. Lysosomes as dynamic regulators of cell and organismal homeostasis. Nat Rev Mol Cell Biol. (2020) 21:101–18. doi: 10.1038/s41580-019-0185-4, PMID: 31768005

[82] Scheper GC van der Knaap MS Proud CG. Translation matters: protein synthesis defects in inherited disease. Nat Rev Genet. (2007) 8:711–23. doi: 10.1038/nrg2142, PMID: 17680008

[83] Sambri I. Lysosomal dysfunction disrupts presynaptic maintenance and restoration of presynaptic function prevents neurodegeneration in lysosomal storage diseases. EMBO Mol Med. (2017) 9:112–32. doi: 10.15252/emmm.201606965, PMID: 27881461 PMC5210158

[84] Zhai B Sun X-Y. Mechanisms of resistance to sorafenib and the corresponding strategies in hepatocellular carcinoma. World J Hepatol. (2013) 5:345–52. doi: 10.4254/wjh.v5.i7.345, PMID: 23898367 PMC3724962

[85] Wang Y. A systematic review and meta-analysis of immune-related adverse events of anti-PD-1 drugs in randomized controlled trials. Technol Cancer Res Treat. (2020) 19:1533033820967454. doi: 10.1177/1533033820967454, PMID: 33084525 PMC7588773

[86] Kim JM Chen DS. Immune escape to PD-L1/PD-1 blockade: seven steps to success (or failure). Ann Oncol. (2016) 27:1492–504. doi: 10.1093/annonc/mdw217, PMID: 27207108

[87] Perez-Riverol Y. The PRIDE database resources in 2022: a hub for mass spectrometry-based proteomics evidences. Nucleic Acids Res. (2022) 50:D543–52. doi: 10.1093/nar/gkab1038, PMID: 34723319 PMC8728295

