# Supplementary material for: Loss of two-pore channel 2 enhances CD8+ T cell cytotoxicity and directly impairs tumour growth via MAPK axis in HCC
Source: Front Immunol. 2025 Oct 24;16:1668066. doi: 10.3389/fimmu.2025.1668066 (PMC12592050; doi:10.3389/fimmu.2025.1668066)
Supplement: Supplementary file 8 [file DataSheet1.docx]

**Supplementary Methods – pH sensors**

**
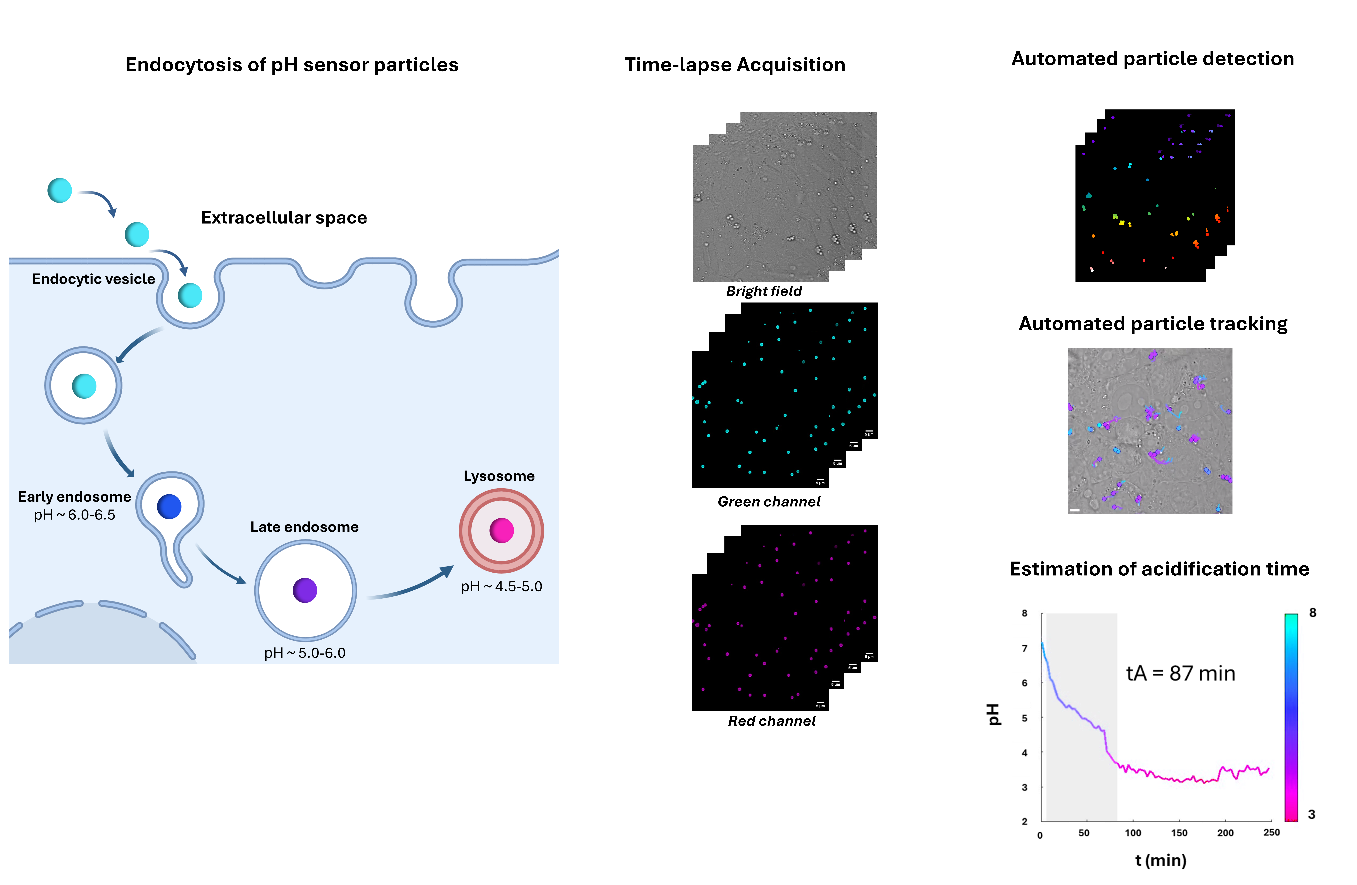
**

**Synthesis and Surface Charge Modification of Ratio metric Optical pH Sensors**

Ratio metric optical pH sensors based on silica (SiO_2_) microparticles were synthesized using a one-pot method adapted and optimized from a previously described protocol ^1^. Briefly, silica seeds were produced via basic hydrolysis adding in the batch reactor the TEOS monomer (200 µL), the NH_4_OH catalyst (760 µL) and the KCl electrolyte (2.8 mg) to an ethanolic aqueous solution (12 mL EtOH, 1.12 mL H_2_O). The reaction was carried out at room temperature under stirring (240 rpm) for 30 minutes to generate monodisperse silica particles. The silica seeds were then functionalized to ratio metric pH sensors using APTES as a linker^2^ and FITC and RBITC as the pH-sensitive and reference dyes, respectively^3^. FITC-APTES and RBITC-APTES conjugates were prepared in parallel by dissolving 0.5 mg of FITC or RBITC in 2 mL of anhydrous EtOH, followed by the addition of 0.13 uL of APTES. The mixtures were stirred at RT for 2 h at 500 rpm. Upon completion of the reactions, the two conjugates were combined, and an additional 336 µL of TEOS was added to promote silica regrowth. The resulting mixture was slowly injected into the silica seed batch reactor using a syringe pump at a controlled flow rate of 0.05 mL/h (RT, 24 h, 240 rpm). The resulting pH sensor particles were collected by centrifugation (2500 rpm, 5 min, RT), washed three times with 50 mL of EtOH and three times with 50 mL of deionized water. The final product was resuspended in EtOH to obtain a 40 mg/mL stock solution and stored in the dark at RT. To promote cellular internalization, the pH sensors were coated with a positively charged polyelectrolyte layer. Specifically, 500 µL of ethanol-based sensor stock (40 mg/mL) was centrifuged, and the pellet was resuspended in 1 mL of poly(allylamine hydrochloride) (PAH, ≈56.000 Da) solution (2 mg/mL, 0.5 M NaCl, pH 6.5) and stirred vigorously (800 rpm) for 15 min. After coating, the sensors were centrifuged again and resuspended again in ethanol (40 mg/mL) for storage and use.

**Characterization of Ratiometric Optical pH Sensors**

The pH sensors were morphologically characterized via scanning electron microscopy (SEM, Sigma 300VP, Carl Zeiss, Germany) under a 5 kV as accelerating voltage, employing a secondary electron detector (SE2) at magnifications of 10,000x, 30,000x, 100,000x. For SEM imaging, a 1 µL of the sensor stock solution was deposited onto a silicon wafer, air-dried at RT overnight and sputter-coated with a 10 nm thick gold layer using a Safematic CCU-010 LV Vacuum Coater. The diameter distribution of the sensors was extracted by drawing circular regions of interest on the acquired SEM images in ImageJ software. The surface charge (zeta potential) of the pH sensors was measured by Dynamic Light Scattering (DLS) (Zetasizer Nano ZS, Malvern Instruments, UK) in deionized water (refractive index in water 1.458, absorption coefficient 0.010, 25°C, 3 min equilibrium time)^4^. Zeta potential data were processed and analysed with the manufacturer’s Zetasizer 7.12 software. The pH sensors were fluorometrically calibrated in DMEM adjusted to pH values within the physiological range (5.0, 5.5, 6.0, 6.5. 7.0, 7.5, 8.0). Reversibility was assessed by performing pH-switching cycles between pH 8.0 and pH 5.0. All fluorescence measurements were conducted using a CLARIOstar® Plus plate reader (CLARIOstar, BMG LABTECH Inc., USA) and analysed using MARS Data Analysis software provided by the manufacturer. Plate reader settings included spectral scans for FITC (λ_ex_= 472 ± 16 nm, λ_em_= 500 ± 10 nm and 700 ± 10 nm) and RBITC (λ_ex_= 550 ± 8 nm, λ_em_ = 570 ± 10 nm and 700 ± 10 nm). Ratiometric fluorescence was determined by calculating the ratio between FITC and RBITC wavefunctions.

**Cytotoxicity Evaluation of Ratiometric pH sensors**

To evaluate the biocompatibility of the ratiometric optical pH sensors, a cytotoxicity assay was performed using the CellTiter-Glo Cell Viability Assay (Promega Corporation, Germany, G7570). Briefly, Hep 3B and RIL175 cells (WT and TPC2 KO) were seeded at a density of 5000 cells per well in 96-well plates and cultured at 37°C in a humidified incubator with 5% CO_2_. After 24 hours, cells were treated with pH sensors at final concentrations of 0.05, 0.1 and 0.2 mg/mL or left untreated as controls. Cell viability was evaluated after 24 and 48 hours by adding an equal volume of CellTiter-Glo reagent to each well. Plates were mixed for 5 minutes on an orbital shaker to ensure efficient cell lysis and ATP extraction. Subsequently, the plates were incubated at room temperature for an additional 25 minutes to equilibrate the luminescent signal, which correlates directly with ATP content and thus viable cell numbers. Luminescence signals were measured using a microplate reader (ClarioStarPlus, BMG Labtech) and untreated cells were used as control. Background luminescence, measured in control wells containing medium and sensors without cells, was subtracted from experimental readings.

**Flow Cytometry Analysis of Intracellular Sensor Uptake**

Intracellular uptake efficiency of pH sensors was measured by flow cytometry. Hep3B and RIL175 cells (WT and TPC2 KO) were seeded at 2x10^5^ cells per well in 6-well plates and cultured at 37°C with 5% CO_2_ for 24 h. Cells were then treated with pH sensors at a concentration of 0.05 mg/mL, intracellular uptake was monitored at defined intervals (0, 15, 30, 60, 120, and 360 minutes). At each time point, cells were washed twice with PBS 1X, detached using trypsin-EDTA solution and resuspended in 300 µL of PBS 1X for immediate flow cytometry analysis (CytoFLEX S, Beckman Coulter, USA). Approximately 20,000 events per sample were acquired and analysed using CytExpert software. Dead cells, debris, and doublets were excluded based on forward scatter and side scatter measurements.

**Immunofluorescence Assay for Lysosomal Co-localization of pH Sensors**

For co-localization analysis of pH sensors within lysosomes, Hep3B and RIL175 cells (WT and TPC2 KO) were seeded onto glass slides at a density of 5x10^4^ cells/well and cultured under standard conditions (37°C, 5% CO_2_) for 24 h. Cells were then fixed with 4% PFA, permeabilized with 0.2% of saponin and incubated overnight at 4°C with anti-LAMP1 primary antibody (at 1:300 dilution; ab278043). After thorough washing, samples were incubated with Alexa Fluor^®^ 647-conjugated secondary antibody (1:400 dilution; Invitrogen A32795) diluted in blocking buffer (2% BSA, 0.1% saponin). Nuclei were counterstained with DAPI (1:1000; Sigma Aldrich, D9542). Coverslips were then mounted using Mowiol mounting media (16% [w/vol] 4−24, EMD Millipore; 30% [vol/vol] glycerol in PBS) and analysed by confocal laser scanning microscopy (CLSM, LSM 980, Carl Zeiss, AG, Germany) using a 63× oil-immersion objective.

**Live Confocal Fluorescence Microscopy and Sensor Calibration**

Prior to live cell imaging, calibration of ratiometric optical pH sensors was performed by CLSM (LSM 980, Carl Zeiss). To this aim, sensors were placed into 8-well chamber slides (IBIDI, Berlin, Germany), pre-coated with fibronectin (0.2 mg/mL), and equilibrated with pH-adjusted cell culture media (pHs 7.0, 6.0, 5.0, 4.0) for 10 minutes. Images were then acquired along the z-axis using CLSMequipped with an Okolab Stage Top Incubator (Okolab s.r.l., Italy) to maintain controlled environmental conditions (5%n CO_2_, 37°C). Following calibration, Hep3B and RIL175 cells (WT and KO) seeded at 5x10 cells per into 8-well chamber slides were incubated with pH sensors (0.05 mg/mL) and placed into the Okolab incubator. Time lapse imaging was set up for 15 hours acquiring *z*-stack images with an acquisition interval of 2 minutes. Images were acquired by using a PlanApochromatic 63X/1.4 oil objective, 1.5× zoom (213.39 µm x 213.39 µm) and resolution of 1024x1024 pixels. Fluorescence emission was collected at λₑₘ = 498–580 nm for FITC (λₑₓ = 488 nm) and λₑₘ = 570–620 nm for RBITC (λₑₓ = 555 nm). Quantitative analysis of the 3D CLSM acquisitions (x,y,z) of the microparticles was done using a modified version of a previously developed algorithm to automatically extract precise pH read-outs^5–7^. Briefly, maximum intensity projections along the z-axis in the red fluorescence channel were segmented using a deep learning-based model implemented in Cellpose^8^. In particular, the pretrained *"cyto"* model was employed to automatically segment individual microsensors. Default settings were used for all parameters, except for the object diameter, which was automatically calibrated to reflect the average size of the microsensors. The resulting labelled images were used as masks to extract mean fluorescence intensity ratios (I_FITC_/I_RBITC_) for each microparticle sensor. Finally, mean fluorescence ratios and their standard deviations were calculated across all analysed sensors to establish the calibration data.

**Particle Detection, Tracking and Dynamic pH Monitoring**

Real-time monitoring of intracellular pH changes over time was achieved by integrating deep learning-based particle segmentation and object tracking algorithms (Figure 1n, main document). Briefly, Cellpose was used to detect individual sensors in each frame, as described in the previous section^8,9^. The resulting labels provided precise spatial coordinates of each sensor throughout the imaging sequence. Particle tracking over time was performed using the Linear Assignment Problem (LAP) algorithm, as implemented in the TrackMate plugin within ImageJ^10,11^. This algorithm accurately links segmented particles across consecutive frames based on minimizing a global cost function, considering particle displacement, intensity, and object size variations. Subsequently, fluorescence intensity ratios (green-to-red channel) were computed frame-by-frame for each tracked particle using custom scripts in MATLAB R2024a, enabling time-resolved monitoring of fluorescence changes. Applying the previously obtained calibration curve to these intensity ratios allowed the conversion of fluorescence data into quantitative pH values, enabling detailed quantitative and temporal assessment of pH dynamics across the different cell lines (**Supplementary Videos 1–5**). The resulting temporal pH curves were visually inspected to classify three distinct event types:

- Outer Event (OE): The sensor remains extracellular throughout the entire observation period, detecting the pH of the surrounding microenvironment without being internalized by the cell.
- Uptake Event (UE): The sensor initially resides outside the cell, measuring the extracellular pH. It is subsequently internalized, enabling the detection of pH changes during the transition from the extracellular to the intracellular environment.
- Inner Event (IE): The sensor remains intracellular for the full duration of observation, continuously monitoring the pH within the cell.

**Quantitative Estimation of Acidification Time**

To quantitatively estimate the acidification time, we specifically analysed the uptake events. During these events, sensors transitioned from cyan upon initial cell contact to deep blue upon cellular internalization, and ultimately to magenta upon complete internalization, reflecting progressive environmental acidification. This colour transition results from pH-dependent fluorescence quenching of FITC and the stable fluorescence of RBITC under acidic conditions, characteristic of endo-lysosomes compartments. For each pH time curve associated with uptake events, isotonic regression (IR)^12,13^ was applied to determine the optimal non-increasing curve that best fits the input data. Subsequently, introducing λ as a normalized free parameter defined by:

$$\lambda=\frac{{pH}_{iso} \left( t \right)-\min{pH}_{iso}}{(\max{pH}_{iso}-\min{pH}_{iso} )}$$

allowed the identification of specific time points corresponding to fractions of the observed pH decrease. The acidification time (Δτ) was then calculated as the time interval required to transition between 90% (λ=0.1) and 10% (λ=0.9) of the pH drop:

$$\Delta\tau=|t \left( \lambda=0.1 \right)-(t \left( \lambda=0.9 \right)|$$

References

1. Iuele, H. *et al.* Optical ratiometric silica pH sensors unveil active transport and subcellular particle localization in protoplasts. *Nano Research* **18,** 94907201; 10.26599/NR.2025.94907201 (2025).

2. Iuele, H. *et al.* Facile One Pot Synthesis of Hybrid Core-Shell Silica-Based Sensors for Live Imaging of Dissolved Oxygen and Hypoxia Mapping in 3D Cell Models. *ACS applied materials & interfaces*; 10.1021/acsami.4c08306 (2024).

3. Siciliano, A. C. *et al.* A 3D Pancreatic Cancer Model with Integrated Optical Sensors for Noninvasive Metabolism Monitoring and Drug Screening. *Advanced healthcare materials* **13,** e2401138; 10.1002/adhm.202401138 (2024).

4. Ojeda-Mendoza, G. J., Contreras-Tello, H. & Rojas-Ochoa, L. F. Refractive index matching of large polydisperse silica spheres in aqueous suspensions. *Colloids and Surfaces A: Physicochemical and Engineering Aspects* **538,** 320–326; 10.1016/j.colsurfa.2017.10.088 (2018).

5. Rizzo, R. *et al.* A pH-sensor scaffold for mapping spatiotemporal gradients in three-dimensional in vitro tumour models. *Biosensors & bioelectronics* **212,** 114401; 10.1016/j.bios.2022.114401 (2022).

6. Rizzo, R. *et al.* pH-sensing hybrid hydrogels for non-invasive metabolism monitoring in tumor spheroids. *Materials today. Bio* **20,** 100655; 10.1016/j.mtbio.2023.100655 (2023).

7. Onesto, V. *et al.* Probing Single-Cell Fermentation Fluxes and Exchange Networks via pH-Sensing Hybrid Nanofibers. *ACS nano* **17,** 3313–3323; 10.1021/acsnano.2c06114 (2023).

8. Stringer, C., Wang, T., Michaelos, M. & Pachitariu, M. Cellpose: a generalist algorithm for cellular segmentation. *Nature methods* **18,** 100–106; 10.1038/s41592-020-01018-x (2021).

9. Stringer, C. & Pachitariu, M. Cellpose3: one-click image restoration for improved cellular segmentation. *Nature methods* **22,** 592–599; 10.1038/s41592-025-02595-5 (2025).

10. Jaqaman, K. *et al.* Robust single-particle tracking in live-cell time-lapse sequences. *Nature methods* **5,** 695–702; 10.1038/nmeth.1237 (2008).

11. Munkres, J. Algorithms for the Assignment and Transportation Problems. *Journal of the Society for Industrial and Applied Mathematics* **5,** 32–38; 10.1137/0105003 (1957).

12. Best, M. J. & Chakravarti, N. Active set algorithms for isotonic regression; A unifying framework. *Mathematical Programming* **47,** 425–439; 10.1007/BF01580873 (1990).

13. Chandra, A. *et al.* Fully Automated Computational Approach for Precisely Measuring Organelle Acidification with Optical pH Sensors. *ACS applied materials & interfaces* **14,** 18133–18149; 10.1021/acsami.2c00389. (2022).
