# Supplementary material for: Loss of two-pore channel 2 enhances CD8+ T cell cytotoxicity and directly impairs tumour growth via MAPK axis in HCC
Source: Front Immunol. 2025 Oct 24;16:1668066. doi: 10.3389/fimmu.2025.1668066 (PMC12592050; doi:10.3389/fimmu.2025.1668066)
Supplement: Supplementary file 9 [file DataSheet2.docx]

***Supplementary Material***

**
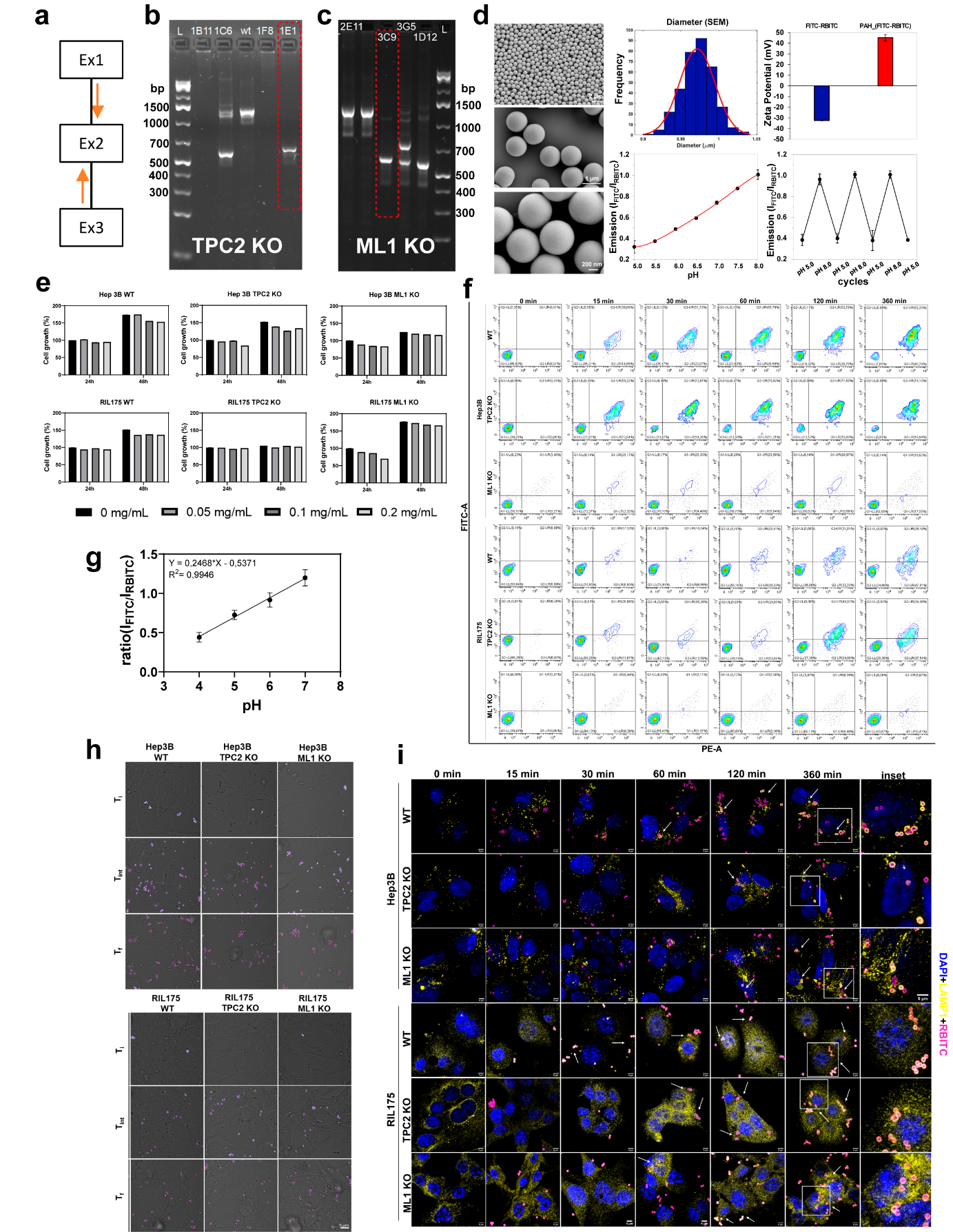
**

**Figure S1: Lysosomal pH sensor measurements.**

**(a)** Deletion of TPCN2 and MCOLN1 in Hep3B resulted in deletion of exon 2. **(b)** Successful deletion was confirmed with gel electrophoresis and Sanger sequencing. Statistical significance was assessed by unpaired students t-test ** p < 0.01. **(d**) Morphological characterization of positively charged pH sensor microparticles. SEM micrographs of pH sensor microparticles acquired at increasing magnifications (10,000x, 30,000x, 100,000x). Diameter distribution of the microparticles as determined from SEM image analysis. ζ-potential measurements showing surface charge of the microparticles before and after coating with a PAH polyelectrolyte layer. Spectrofluorimetric calibration curve of the sensor particles obtained by plotting fluorescence emission ratios vs known pH values. Assessment of sensor reversibility through repeated cycles of pH 5.0 and pH 8.0. (n ≥ 3). **(e)** Cell viability following exposure to ratio metric pH-sensitive microparticles at different concentrations for 24 and 48 h. No significant cytotoxicity was observed, confirming the biocompatibility of the pH sensors (n=3). **(f)** Representative flow cytometry dot plots illustrating time-dependent internalization of pH sensors in WT and TPC2 KO cells. **(g)** Corresponding calibration curve plotting the fluorescence intensity ratio (FITC/RBITC) against pH, demonstrating a robust and linear response across the tested range. **(h)** Representative CLSM micrographs of WT and TPC2 KO Hep3B and RIL175 cells exposed to ratio metric pH sensors, acquired at key time points: before internalization (Ti), during internalization (Tint), and after internalization (Tf), Images were acquired using green (FITC), red (RBITC), and transmission channels, with composite overlays shown. Z-stack step size=0.5 μm; Scale bars, 5 μm. **(i)** Specific lysosomal localization of optical pH sensors in WT and TPC2 KO cells. Representative CLSM micrographs showing the subcellular distribution of pH sensor particles in WT and TPC2 KO cells. The pH sensors (RBITC, magenta) co-localize with the lysosomal marker LAMP1 (yellow), while nuclei were counterstained with DAPI (blue). Insets show selected regions of clear co-localization by prominent light pink signal in merged. Scale bars 5 µm. (j) (k) Acidification times across different cell lines. Outliers were determined via ROUT.

**
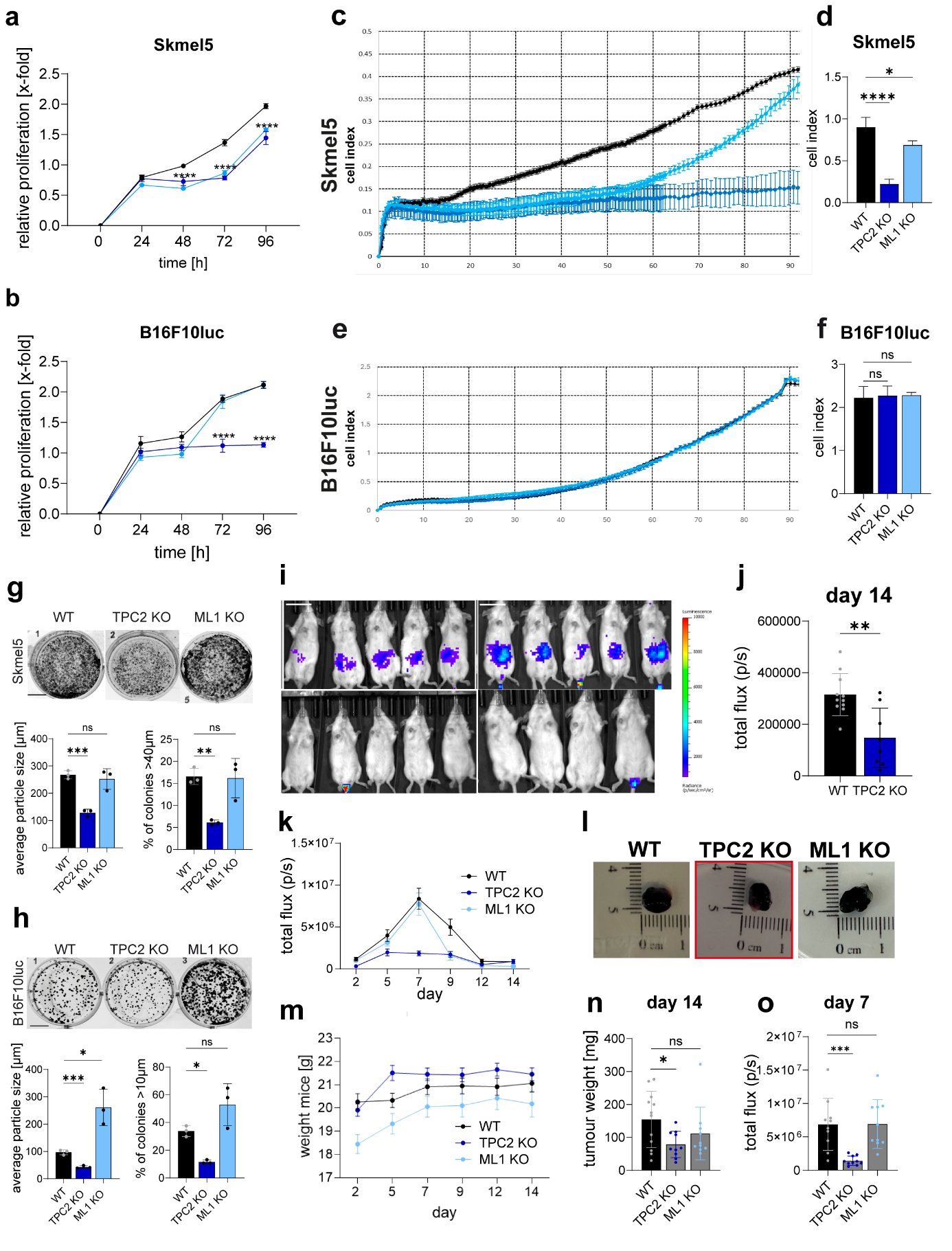
**

**Figure S2: Loss of TPC2 results in reduced cancer proliferation.**

**(a,b)** Cancer cell proliferation monitored via CellTiter-Blue assay over 96h (n=5) in Skmel5 and B16F10luc cells. **(c-f)** XCelligence impedance measurements over 92h. Cell index and calculated doubling time from impedance measurements of Skmel5 **(d)** and RIL175 **(f)** cells. **(g,h)** Colony formation in TPC2 KO cells significantly reduced over 5 days (n=3). Scale bar 1 cm. **(i)** IVIS images of all mice on day 14 (n=10 per group). Scale bar 5 cm. One mouse in TPC2 KO cohort had to be sacrificed, due to illness. RIL175 TPC2 KO cells disseminated less in periphery organs than WT cells. **(j)** Total flux on day 14 (endpoint). **(k)** Tumour size was indirectly measured non-invasively via IVIS imaging over 14 days. Graphs show a significant reduction in tumour growth in TPC2 KO tumours. Outliers were identified with ROUT, Q=1%. **(l)** Images of ex vivo RIL175 tumours after endpoint day 14. **(m)** Body weight of all mice included in the ectopic melanoma experiment. (n) Tumour weights after endpoint day 14. **(o)** Total flux on day 7. Statistical significance was assessed by two-way ANOVA Dunnett's multiple comparisons test or one-way ANOVA Dunnett's multiple comparisons test * p < 0.05, ** p < 0.01, *** p < 0.001, **** p<0.0001, ns = not significant.


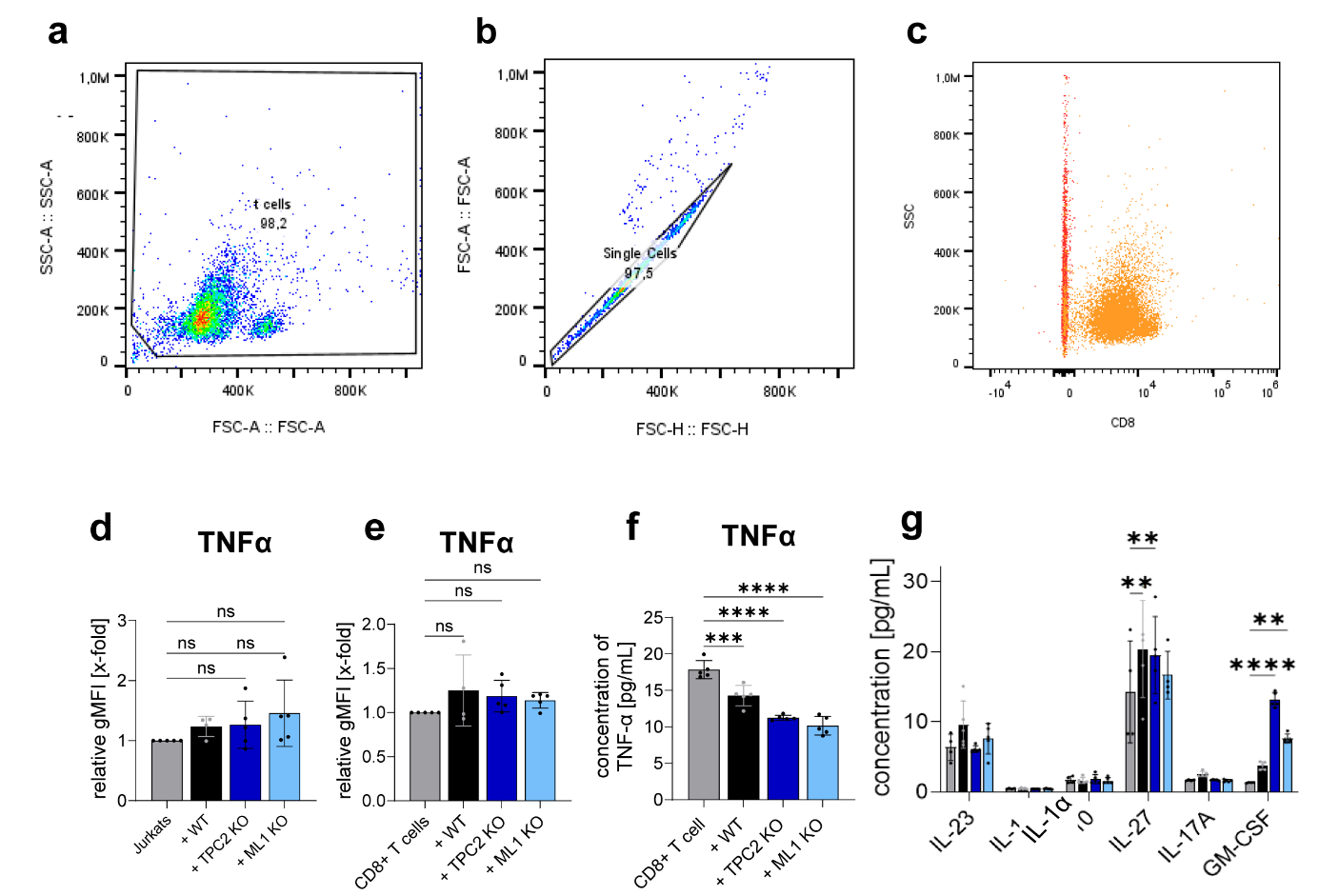


**Figure S3: Gating strategy for all flow cytometer measurements.**

**(a)** Living cell population was determined over SSC/FCS. Gate was set to exclude cell debris. **(b)** Subsequently single cell population was determined via FSC:A/FCS:H. gMFIs of single cell populations were determined for the respective lasers depending on the fluorescence tag. **(c)** CD8^+^ T cells isolated from spleens were checked for their CD8 expression. Isotype control in red, CD8^+^ T cells orange. **(d,e)** TNF-α levels in co-culturing assay (n=5). Concentration in supernatant significantly reduced **(f)**, whilst TNF-α not significantly changed in T cells (d,e). **(g)** Concentration of further cytokines altered in supernatant of CD8^+^ T cell/RIL175 cell co-culture determined via Legendplex assay. All other cytokines left from the 13plex panel remained unaltered (n=5). Statistical significance was assessed by One-way ANOVA Dunnett's multiple comparisons test ** p < 0.01, **** p<0.0001, ns = not significant.


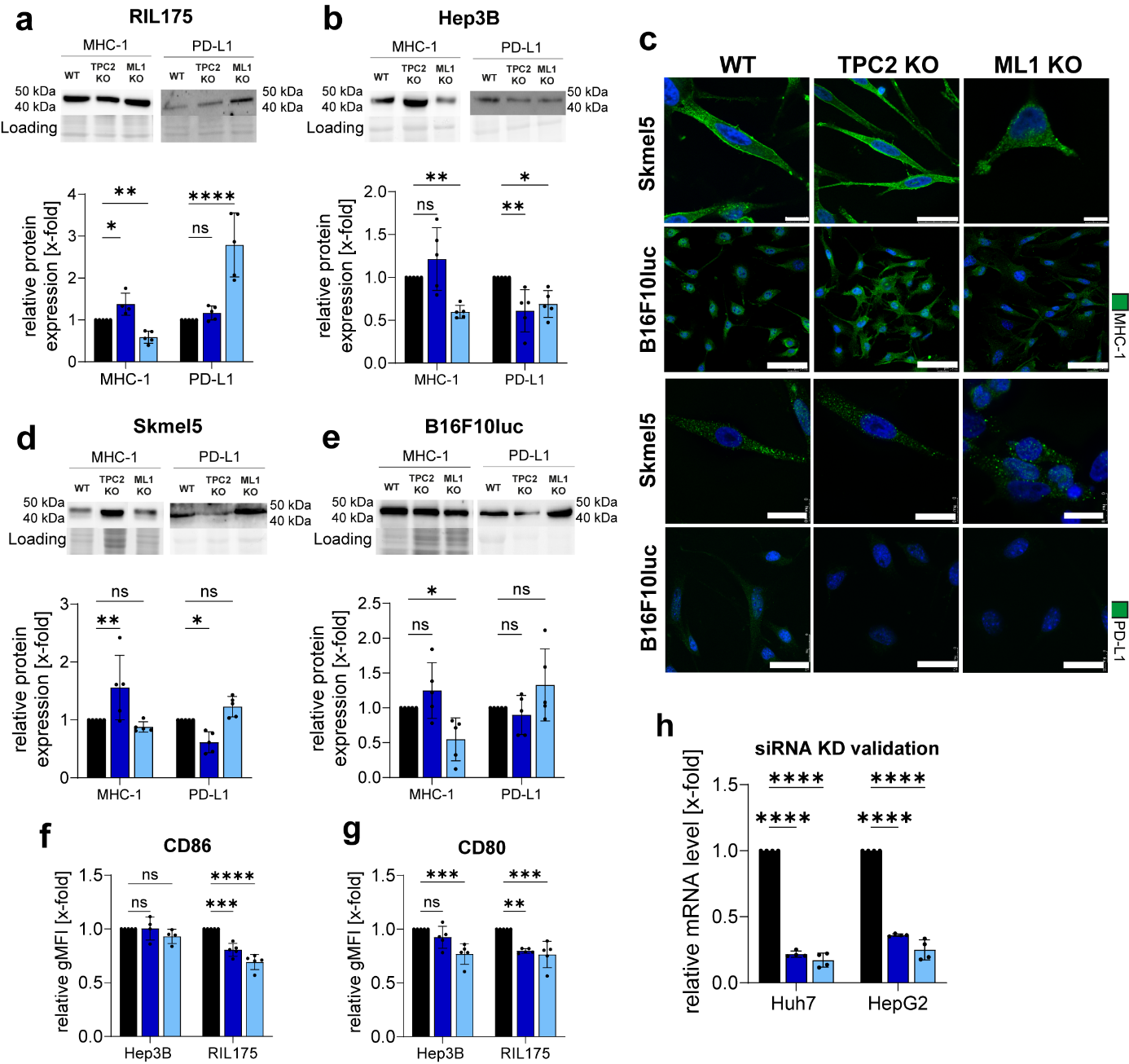


**Figure S4: TPC2 KO increases MHC-I and decreases PD-L1 levels.**

**(a,b)** Relative protein levels of MHC-I (MHC-1) and PD-L1 in total protein lysates of HCC cell lines (n=5). **(c)** Confocal images of MHC1 and PD-L1 on cell surface, nuclei stained with Hoechst (blue). Representative images are shown (n=3). Scale bar 25µm. **(d,e)** Relative protein levels of MHC-I (MHC-1) and PD-L1 in total protein lysates of melanoma cell lines (n=5). **(f****,g)** Cell surface levels of CD86 (f) and CD86 (g) on cancer cells determined via flow cytometry (all n=5). All images/Blots are representative. **(h)** siRNA knockdown validation via RT-qPCR. GAPDH served as a housekeeping gene (n=4). Statistical significance was assessed by One-way ANOVA Dunnett's multiple comparisons test * p < 0.05, ** p < 0.01, *** p < 0.001, **** p<0.0001, ns = not significant.

**
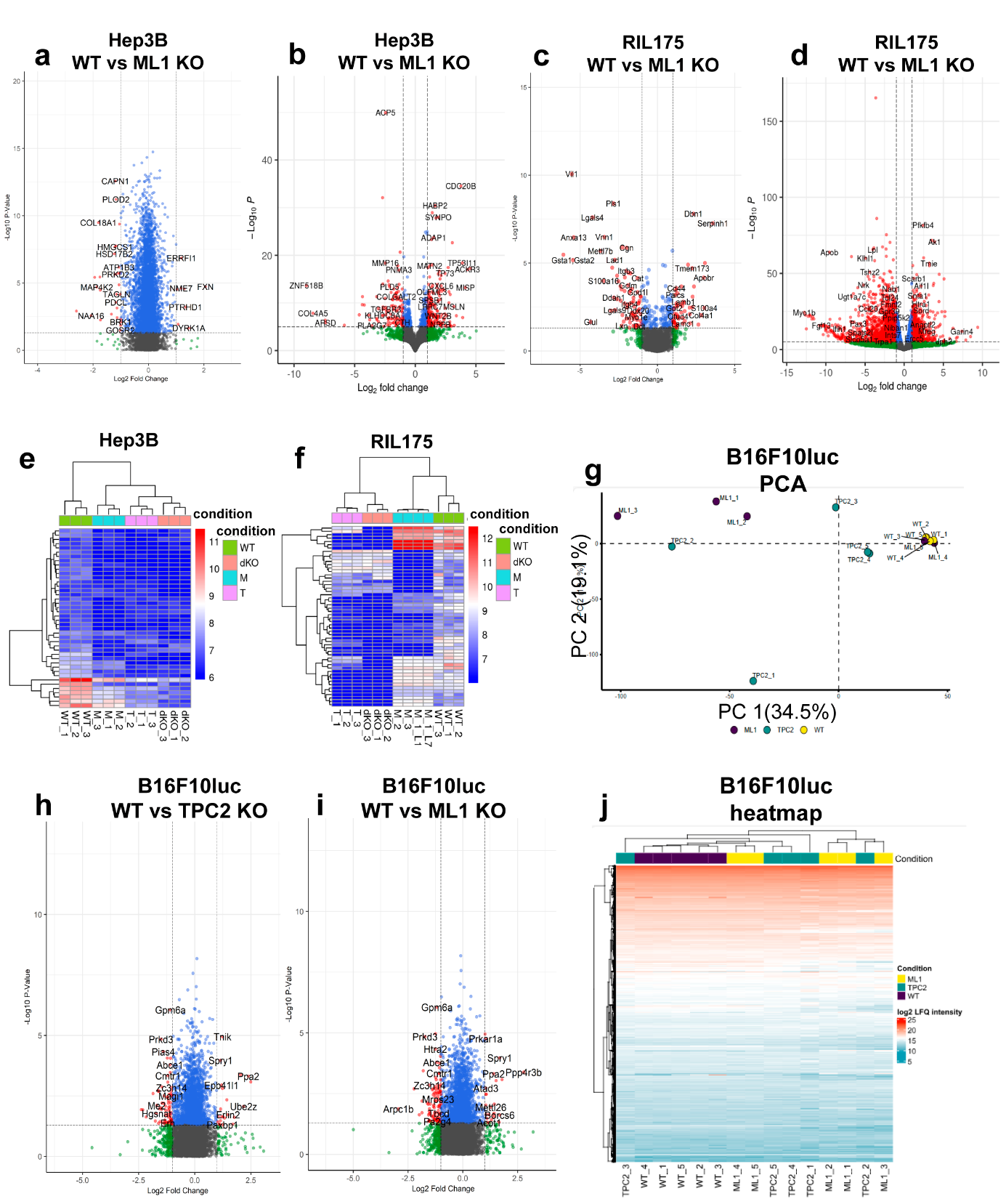
**

**Figure S5: Proteome data on WT vs ML1 KO and of B16F10luc.**

**(a,b)** Volcano plots of Hep3B WT vs ML1 KO proteomic **(a)** and RNA-seq **(b)** data displaying all differentially abundant proteins (red). Filtering was based on p< 0.05 and Log2 fold change > 1. Log2 fold change >1 = more abundant proteins, log2 fold change <1 less abundant proteins. Proteomics total variables =7577, RNA-seq total variables =27106, **(c,d)** Volcano plots of RIL175 WT vs ML1 KO proteomic **(c)** and RNA-seq **(d)** data displaying all differentially abundant proteins (red). Filtering was based on p < 0.05 and Log2 fold change > 1. Log2 fold change >1 = more abundant proteins, log2 fold change <1 less abundant proteins. Proteomics total variables =1960, RNA-seq total variables =24321. **(e,f)** Heat map demonstrating label-free quantification intensities for all genes in all three RNA-seq experimental setups with dendrogram-based grouping according to experimental conditions. **(g)** B16f10luc PCA showing sample-wise grouping according to experimental conditions from proteome analysis. **(h,i)** Volcano plots of B16F10luc WT vs TPC2 KO **(h)** and WT vs ML1 KO **(i)** proteomic data displaying all differentially abundant proteins (red). Filtering was based on p< 0.05 and Log2 fold change > 1. Log2 fold change >1 = more abundant proteins, log2 fold change <1 less abundant proteins. Proteomics total variables =7227. **(j)** B16F10luc heat map demonstrating label-free quantification intensities for all genes in all three experimental setups with dendrogram-based grouping according to experimental conditions.


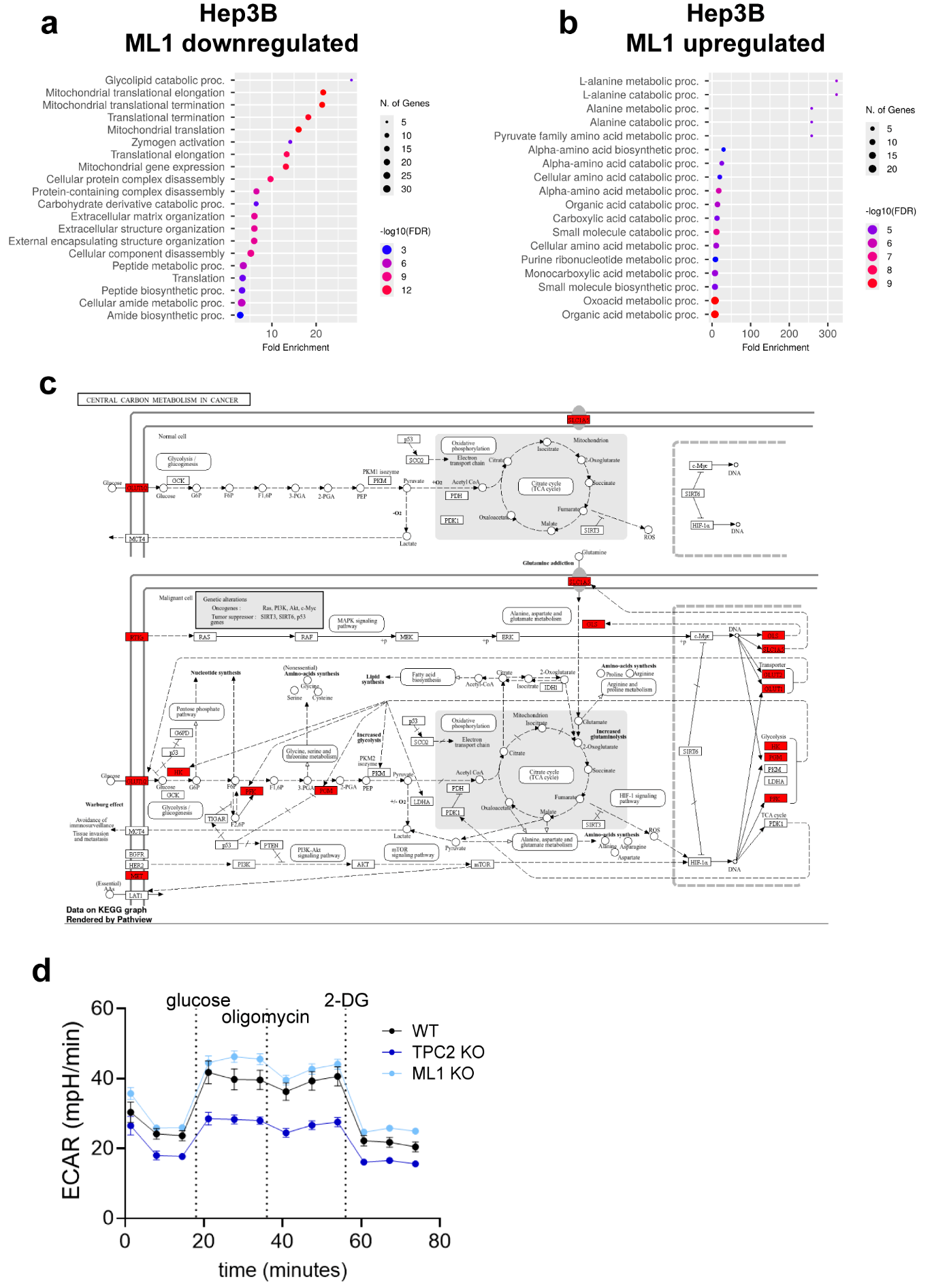


**Figure S6: GSEA of ML1 KO vs WT proteome data.**

**(a-c)** GSEA of proteome data of Hep3B cells displaying top 20 altered BP pathways. ML1 KO vs. WT showed reduced mitochondrial activity and extracellular matrix organisation **(a)**, whilst catabolic processes were upregulated **(b)**. **(c)** KEGG pathway analysis show significant downregulation of proteins regarding glucose transporter (GLUT1/2) and RTKs. Affected proteins are highlighted in red. Dotplots and KEGG graph was modelled via ShinyGOv0.76 and KEGG graph rendered by Pathview. **(d)** ECAR measurements of Hep3B cells using Seahorse assay (n=3).


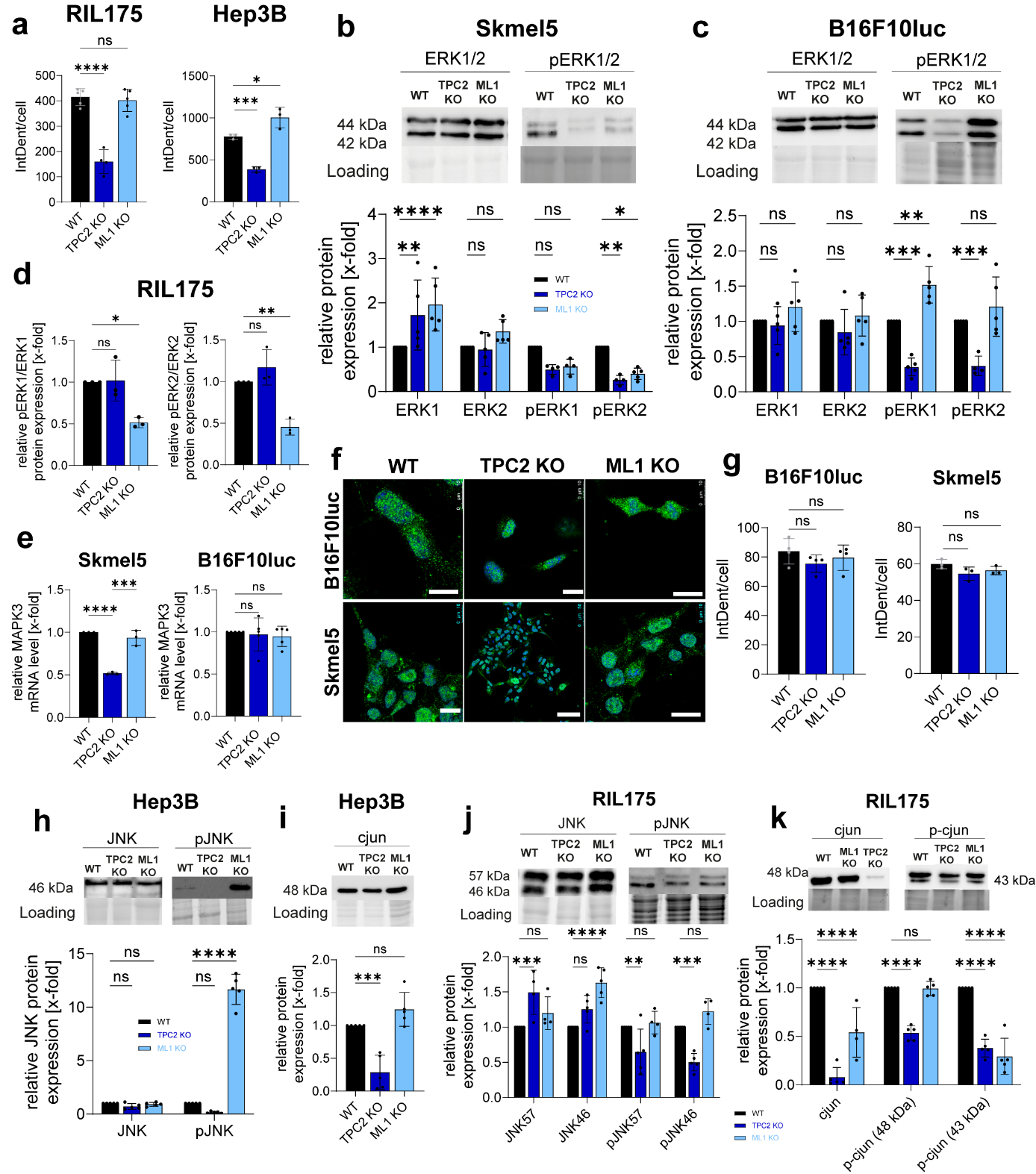


**Figure S7: Altered ERK1/2 and JNK protein expression upon loss of TPC2.**

**(a**) Fluorescence intensities were measured by ImageJ and normalized to the number of cells per image. **(b,c)** Relative protein levels of ERK1/2 and pERK1/2. Images/Blots are representative. **(d)** Quantification of relative protein expression of phosphorylated ERK1/2 to total ERK1/2. Images/Blots are representative. **(e)** RT-qPCR of MAPK3 (ERK1). Actin served as housekeeping gene. (Skmel5 n=3, B16F10luc n=5). **(f)** Confocal images of ERK1/2. Nuclei stained with Hoechst (blue) and quantification. Scale bars 10µm. Representative images shown (all n=3). **(g)** Fluorescence intensities were measured by ImageJ and normalized to the number of cells per image. **(h,j)** Relative protein levels of JNK and pJNK. Images/Blots are representative. **(i,k)** Relative protein levels of JNK downstream target cjun and phosohorylated cjun **(k)**. **(j)** All n=5. Statistical significance was assessed by One-way ANOVA Dunnett's multiple comparisons test * p < 0.05, ** p < 0.01, *** p < 0.001, **** p<0.0001, ns = not significant.

**Supplementary Video 1:** Time-lapse visualization of the trajectory of a single microparticle in Hep3B WT cells, colour-coded according to local pH changes over time (cyan= neutral pH; magenta = acidic pH). The size of the scale bar corresponds to 5 μm.

**Supplementary Video 2:** Time-lapse visualization of the trajectory of a single microparticle in Hep3B TPC2 KO cells, colour-coded according to local pH changes over time (cyan= neutral pH; magenta = acidic pH). The size of the scale bar corresponds to 5 μm.

**Supplementary Video 3:** Time-lapse visualization of the trajectory of a single microparticle in Hep3B ML1 KO cells, colour-coded according to local pH changes over time (cyan= neutral pH; magenta = acidic pH). The size of the scale bar corresponds to 5 μm.

**Supplementary Video 4:** Time-lapse visualization of the trajectory of a single microparticle in RIL175 WT cells, colour-coded according to local pH changes over time (cyan= neutral pH; magenta = acidic pH). The size of the scale bar corresponds to 5 μm.

**Supplementary Video 5**: Time-lapse visualization of the trajectory of a single microparticle in RIL175 TPC2 KO cells, colour-coded according to local pH changes over time (cyan= neutral pH; magenta = acidic pH). The size of the scale bar corresponds to 5 μm.

**Supplementary Video 6**: Time-lapse visualization of the trajectory of a single microparticle in RIL 175 ML1 KO cells, colour-coded according to local pH changes over time (cyan= neutral pH; magenta = acidic pH). The size of the scale bar corresponds to 5 μm.

**Supplementary Video 7**: Time-lapse visualization of the trajectory of multiple microparticles, colour-coded according to local pH changes over time (cyan= neutral pH; magenta = acidic pH). The size of the scale bar corresponds to 5 μm.

**Table S1: Primary and secondary antibodies**

| Antibodies | | |
| --- | --- | --- |
| Reagent | **Source** | **Catalogue Number** |
| p44/42 MAPK (Erk1/2) | Cell Signaling Technology | 9102S |
| phospho- p44/42 MAPK (ERK1/2) | Cell Signaling Technology | 9106 |
| JNK | Cell Signaling Technology | 9252 |
| JNK-phospho (T183/Y185) | Cell Signaling Technology | 9251 |
| c-Jun (60A8) | Cell Signaling Technology | 9165T |
| Phospho-c-Jun (Ser73) | Cell Signaling Technology | 3270T |
| MHC Class I | Cell Signaling Technology | 76828 |
| PD-L1 (D5V3B) | Cell Signaling Technology | 64988 |
| PD-L1 (E1L3N®) | Cell Signaling Technology | 13684S |
| PD-L1/CD274 | Proteintech | 28076-1-AP |
| CD8α (D4W2Z) | Cell Signaling Technology | 98941S |
| CD80 anti-human (FITC) | BioLegend | 305206 |
| CD86 anti-human (APC) | BioLegend | 3743206 |
| CD80 anti-mouse (APC) | BioLegend | 104714 |
| CD86 anti-mouse (PE) | BioLegend | 159204 |
| H-2Db Antibody, anti-mouse, PE, REAfinity™ | Miltenyi Biotec B.V. | 130-128-078 |
| APC anti-mouse CD274 (B7-H1, PD-L1) | BioLegend | 124311 |
| FITC anti-human CD274 (B7-H1, PD-L1) | BioLegend | 393606 |
| APC anti-human HLA-A,B,C | BioLegend | 311410 |
| IFN-γ Antibody, anti-mouse, FITC, REAfinity™ | Miltenyi Biotec B.V | 130-117-780 |
| TNF-α Antibody, anti-mouse, APC, REAfinity™ | Miltenyi Biotec B.V | 130-123-277 |
| CD8a Antibody, anti-mouse, PE, REAfinity™ | Miltenyi Biotec B.V | 130-123-781 |
| CD8 Antibody, anti-human, FITC, REAfinity™ | Miltenyi Biotec B.V | 130-110-677 |
| TNF-α Antibody, anti-human, APC | Miltenyi Biotec B.V | 130-117-531 |
| IFN-γ Antibody, anti-human, FITC, REAfinity™ | Miltenyi Biotec B.V | 130-114-023 |
| LAMP1 | Abcam | ab278043 |
| Atezolizumab | MedChemExpress | HY-P9904 |
| Nivolumab | MedChemExpress | HY-P9903 |
| Isotype control mouse (FITC) | \| BD Pharmingen \| \| --- \| | 555742 |
| Isotype control mouse (APC) | BioLegend | 981806 |
| PE anti-rat IgG2a | BioLegend | 407508 |
| APC Armenian Hamster IgG | BioLegend | 400912 |
| HRP, Goat- Anti-Mouse IgG1 | abcam | ab97240 |
| HRP, Goat-Anti-Rabbit, normal | dianova | 111-035-144 |
| Alexa Fluor 488, goat anti-rabbit IgG (H+L) | Molecular Probes / Invitrogen | A11008 |
| Alexa Fluor® 647, donkey anti-rabbit IgG (H+L) | Molecular Probes / Invitrogen | A32795 |

**Table S2: Oligonucleotide sequences**

| Oligonucleotides | | |
| --- | --- | --- |
| Name | **Sequence (‘5-…..-3’)** | **Source** |
| hTPCN2 sgRNA A | G ACC CAC CCG GGA CCT AGA AT | Yuan et al., 2022, PMID: 3518320 |
| hTPCN2 sgRNA A-rc | ATT CTA GGT CCC GGG TGG GTC | Yuan et al., 2022, PMID: 3518320 |
| hTPCN2 sgRNA F | GAG CGT GGA CAC TCG TGA CT | Yuan et al., 2022, PMID: 3518320 |
| hTPCN2 sgRNA F-rc | AGT CAC GAG TGT CCA CGC TC | Yuan et al., 2022, PMID: 3518320 |
| hMCOLN1 sgRNA A | GGG TCC CAG CTA CTA ACT AC | This paper |
| hMCOLN1 sgRNA A-rc | GTA GTT AGT AGC TGG GAC CC | This paper |
| hMCOLN1 sgRNA C | GAA AAG GGA CCC AAT TGT CC | This paper |
| hMCOLN1 sgRNA C-rc | GGA CAA TTG GGT CCC TTT TC | This paper |
| hTPC2 ex2sp_F3 | CCGAGGCACTTACCTGGTTT | This paper |
| hTPC2 ex2sp_R1 | TGAAAACACAGGAGCCAGCA | This paper |
| hML1 ex2sp_R1 | TGGGGTGTATCTCCCTCAGG | This paper |
| hML1 ex2sp_F4 | ACCAGCCCCTGTGATCAATG | This paper |
| hActin (FW) | CCAACCGCGAGAAGATGA | This paper |
| hActin (RV) | CCAGAGGCGTACAGGGATAG | This paper |
| mActin (FW) | CCACCATGTACCCAGGCATT | This paper |
| mActin (RV) | AGGGTGTAAAACGCAGCTCA | This paper |
| hMAPK3 (FW) | AGAAGAGCCGAGATCAGGGT | This paper |
| hMAPK3 (RV) | ACCATGGGGCAAATACTGGG | This paper |
| mMAPK3 (FW) | CCTTGTTGCCCTTGCTTGAC | This paper |
| mMAPK3 (RV) | AAATCCTGGCATTGGGAGGG | This paper |
| hPDL1 (FW) | CCCCAACCTGAATGAGCCAT | This paper |
| hPDL1 (RV) | GGGGTGCAGCTGTCATTAGT | This paper |
| mPDL1 (FW) | TTGTTGGACCTGTTGGCCTT | This paper |
| mPDL1 (RV) | CGGCTCACAAGGAACAGTCT | This paper |

**Table S3: Proteome data Hep3B TPC2 KO vs WT - High level GO category**

| N | High level GO category | Genes |
| --- | --- | --- |
| 126 | Regulation of biological quality | CPS1 ALAS1 VIM SLC4A7 CP HERPUD1 ATP1B3 PFN2 ATP2B1 SCARB1 IGF2BP2 CA12 LMAN1 PLXNA2 CEACAM1 AFP APOB HSD17B2 TESC ICAM1 TF SH3GLB1 SCD PALMD TBL1X ATP11C FMR1 COTL1 EEF2K SLC39A14 SULT2A1 LSR MET LOX HMGCR PRKAR2A GLS ADGRL2 SLC2A1 TTR FOXO3 GPAM GOT1 SCPEP1 C4BPB ALKBH7 WNK4 PPAT SPART DAGLA ANXA1 TBRG4 ARRB1 PNPT1 MTTP MPC2 AGTR1 LYAR SNCA NAF1 IQGAP2 GAS2 SESN3 HNMT FOXO1 HOMER1 PFKM HK1 NPTN FRRS1 ATP2B2 FMNL2 APOA2 SV2A HK2 THEM4 AZGP1 LRP5 TPCN2 PLPP3 FLVCR1 SLC2A2 SLC9B2 ITGA2 NDUFAF2 FABP5 NAV2 PCSK9 CNBP ELOVL6 FGG FGA FGB SLC25A33 GPHN PAH BSG ADH6 GLRX SLC29A2 A2M BNIP3 CTNNBIP1 ERN1 F2 BHLHA15 PYCR1 PROS1 ROBO2 ADH1A LIN28B DDI2 ENPP1 ADH4 HSD17B11 ASPH DMD TRIM71 ITGA1 GPX1 APOM PINX1 GATC DNAJA3 SERPINF2 |
| 98 | Response to stress | NPC1L1 CPS1 ALAS1 VIM MAP2K3 HERPUD1 PDIA5 WIPI1 ATP2B1 SCARB1 LMAN1 ARHGEF10L CEACAM1 ICAM1 TFAP4 TF SH3GLB1 MIEF1 FMR1 COTL1 EEF2K MET MAN1A1 LOX HMGCR THBS4 FAM162A PRKAR2A ERRFI1 LGALS8 CD46 SLC2A1 ARG1 FOXO3 GPAM GOT1 TNFRSF10B LIAS C4BPA C4BPB ALKBH7 SDF2L1 SLC38A2 DAGLA ANXA1 NT5E TANK ARRB1 PNPT1 DUSP6 AGTR1 AHSG LYAR SNCA SIGMAR1 SESN3 HMGA2 HNMT FOXO1 HK1 APOA2 HK2 PLPP3 AK4 EXTL2 FABP1 ITGA2 NOLC1 POLR3D PCSK9 CD14 MTSS1 FGG FGA FGB MST1 A2M BNIP3 CTNNBIP1 ERN1 DCTPP1 F2 BHLHA15 SSTR2 RELL1 PLCB1 PYCR1 TSPYL2 PROS1 PRMT6 DMD C2 GPX1 C4B_2 ADSL CFB DNAJA3 SERPINF2 |
| 95 | Catabolic process | CPS1 VIM HERPUD1 BCAT1 OAT WIPI1 SCARB1 IGF2BP2 CEACAM1 BCKDHB HAL APOB SH3GLB1 GCAT TIMP3 TBL1X FMR1 FAH SULT2A1 MET ALDOC ATG2A MAN1A1 HMGCR ARSB PFKFB4 GLS LGALS8 ARG1 FOXO3 GDA ALDH6A1 GOT1 KBTBD7 LYPLA1 SARDH C4BPA C4BPB MTRR MCEE PPAT SDF2L1 TST SCLY DAGLA NT5E TBRG4 ARRB1 PNPT1 AMDHD1 SNCA NAF1 AUH SESN3 HNMT FOXO1 MAT1A INPP1 PFKM PELO CARNMT1 HK1 APOA2 HK2 ALDH4A1 LRP5 TPCN2 FABP1 FABP5 PGAM2 NAALADL1 PCSK9 PGM2 PAH AGXT BNIP3 ERN1 DCTPP1 PLCB1 LIN28B ACADSB DDI2 ENPP1 VPS13A ADH4 HIBCH HSD17B11 NUDT16 TRIM71 GPX1 BCKDHA PRODH2 PKLR |
| 94 | Regulation of response to stimulus | MAP2K3 HERPUD1 ATP2B1 PAG1 CEACAM1 APOB ICAM1 SORBS1 TIMP3 TBL1X GPR143 FMR1 SLC39A14 FGL1 MET TSPAN14 ARFGEF3 LOX HMGCR THBS4 PRKAR2A ERRFI1 CD46 ARG1 FOXO3 GPAM GOT1 KBTBD7 TNFRSF10B C4BPA C4BPB MTUS1 SPART SLC38A2 DAGLA ANXA1 NT5E TANK ARRB1 PNPT1 DUSP6 AGTR1 AHSG LYAR SNCA RGS10 GAS2 SESN3 HMGA2 FOXO1 HOMER1 PELO NPTN SKI THEM4 PLPP3 POGLUT1 ITGA2 NDUFAF2 FABP5 CTHRC1 NDRG2 POLR3D PCSK9 CD14 MCC FGG FGA FGB MST1 A2M CTNNBIP1 ERN1 F2 RELL1 PLCB1 PYCR1 TSPYL2 PROS1 ROBO2 NCR3LG1 ENPP1 PRMT6 DMD C2 ITGA1 GPX1 C4B_2 PEG10 CFB VASN DNAJA3 SERPINF2 |
| 86 | Macromolecule localization | NPC1L1 EPHA3 HERPUD1 TRAM2 PPP2R5A ATP1B3 WIPI1 SCARB1 IGF2BP2 LMAN1 TIMM21 CEACAM1 EPB41L2 APOB TESC TF SORBS1 SH3GLB1 MIEF1 ATP11C FMR1 ZFAND1 SQLE LSR TSPAN14 GRPEL1 ZBTB16 CHKA MAN1A1 HMGCR EHBP1 SLC2A1 SPCS2 LYPLA1 ACSL3 ALKBH7 RRBP1 WNK4 TST ANXA1 TBC1D4 ODF2 ARRB1 PNPT1 MTTP MPC2 AGTR1 NAF1 SIGMAR1 SEC24D PFKM HK1 NPTN APOA2 TOMM40L HK2 AZGP1 LRP5 RFTN2 SLC2A2 FABP1 SLC9B2 ITGA2 NDUFAF2 FABP5 NOLC1 VAMP5 PCSK9 MCC PPID FGG FGA FGB GPHN BSG AGXT NECTIN3 F2 MCFD2 ENPP1 VPS13A ASPH DMD APOM PINX1 DNAJA3 |
| 85 | Regulation of signaling | MAP2K3 HERPUD1 ATP1B3 PFN2 ATP2B1 CEACAM1 ICAM1 SORBS1 TIMP3 TBL1X GPR143 FMR1 SLC39A14 MET TSPAN14 ARFGEF3 LOX HMGCR PRKAR2A ERRFI1 CD46 SLC2A1 ARG1 FOXO3 GPAM GOT1 KBTBD7 TNFRSF10B SLC6A6 SPART ANXA1 TANK ARRB1 DUSP6 MPC2 AGTR1 AHSG SNCA RGS10 GAS2 SESN3 HMGA2 FOXO1 HOMER1 PFKM PELO NPTN ATP2B2 SKI THEM4 LRP5 PLPP3 POGLUT1 SLC2A2 SLC9B2 ITGA2 NDUFAF2 FABP5 CTHRC1 NDRG2 PCSK9 CD14 MCC FGG FGA FGB MST1 A2M CTNNBIP1 ERN1 F2 RELL1 PLCB1 TSPYL2 ROBO2 ENPP1 ASPH PRMT6 DMD ITGA1 GPX1 PEG10 VASN DNAJA3 SERPINF2 |
| 84 | Cellular localization | EPHA3 HERPUD1 TRAM2 PPP2R5A ATP1B3 PFN2 WIPI1 LMAN1 TIMM21 CEACAM1 EPB41L2 TESC TF SORBS1 SH3GLB1 TIMP3 MIEF1 GPR143 FMR1 COTL1 ZFAND1 SLC39A14 TSPAN14 ALDOC GRPEL1 ZBTB16 MAN1A1 HMGCR ARSB TTR SPCS2 ARG1 LYPLA1 A1BG ACSL3 DDC TBC1D4 ODF2 GEMIN7 MPC2 EML4 AHSG SNCA IQGAP2 SEC24D HK1 NPTN ZFYVE9 TOMM40L SV2A HK2 LRP5 TPCN2 GYG1 S100P SLC9B2 NDUFAF2 FABP5 NOLC1 VAMP5 PCSK9 PGM2 CD14 FGG FGA FGB GPHN BSG SLC29A2 A2M NECTIN3 F2 MCFD2 BHLHA15 PROS1 FAM3C VPS13A ASPH DMD TXNDC5 PINX1 DNAJA3 SERPINF2 |
| 78 | Response to external stimulus | CPS1 VIM EPHA3 WIPI1 ATP2B1 SCARB1 PLXNA2 CEACAM1 BCKDHB APOB ICAM1 TF FKBP5 SH3GLB1 FMR1 COTL1 MET HMGCS1 LOX HMGCR ARSB THBS4 PRKAR2A LGALS8 ADGRL2 CD46 SLC2A1 ARG1 FOXO3 GPAM GOT1 TNFRSF10B LIAS C4BPA C4BPB ACSL3 MTUS1 SLC38A2 DAGLA ANXA1 NT5E AGTR1 AHSG LYAR SNCA SESN3 HMGA2 FOXO1 HK1 NPTN ITGA2 POLR3D PCSK9 CD14 FGG FGA FGB BSG GNG12 MST1 SLC6A19 A2M BNIP3 DCTPP1 F2 BHLHA15 SSTR2 PROS1 ROBO2 DMD C2 ITGA1 GPX1 C4B_2 ADSL CFB DNAJA3 SERPINF2 |
| 78 | Cellular component biogenesis | EPHA3 TTC17 PFN2 WIPI1 LMAN1 ARHGEF10L TIMM21 APOB DIMT1 DDX18 ICAM1 TFAP4 SORBS1 SH3GLB1 FMR1 COTL1 EEF2K SQLE SLC1A5 LSR MET NPM3 ATG2A LOX GLS ADGRL2 SLC2A1 ACSL3 ERAL1 COX16 ODF2 ARRB1 PNPT1 WDR12 MTTP GEMIN7 LYAR SNCA NAF1 IQGAP2 UTP23 SIGMAR1 SEC24D MAT1A HOMER1 KCTD15 NPTN APOA2 SV2A ITGA2 NDUFAF2 NOLC1 CENPV MTSS1 TMEM126B PPID FGG FGA FGB SLC25A33 NDUFAF3 CTNNBIP1 NSUN3 MPI MCFD2 PSMG4 GLRX5 UTP11 PSMG1 TSPYL2 ROBO2 LYRM7 NUDT16 DMD TSPAN4 APOM SERPINF2 ASAP3 |
| 78 | Regulation of multicellular organismal process | MAP2K3 ATP1B3 PFN2 ATP2B1 SCARB1 IGF2BP2 PLXNA2 CEACAM1 TESC ICAM1 TF SCD ATP11C FMR1 EEF2K MET ZBTB16 SOX6 LOX HMGCR ARSB THBS4 GLS ERRFI1 ADGRL2 CD46 ARG1 FOXO3 GPAM WNK4 GAMT SPART CYP2J2 ANXA1 ARRB1 DUSP6 AGTR1 AHSG HMGA2 FOXO1 HOMER1 HK1 NPTN ATP2B2 SKI APOA2 HK2 PLPP3 FLVCR1 POGLUT1 SLC9B2 ITGA2 FABP5 NDRG2 POLR3D PCSK9 CD14 ELOVL6 MCC FGG FGA FGB BSG MST1 A2M CTNNBIP1 F2 SSTR2 PLCB1 PROS1 ROBO2 ENPP1 ASPH PRMT6 DMD APOM VASN SERPINF2 |
| 76 | Immune system process | ALAS1 VIM ATP1B3 TPD52 PAG1 CEACAM1 APOB TESC ICAM1 TF ATP11C COTL1 FGL1 TSPAN14 ALDOC ZBTB16 LOX ARSB THBS4 PRKAR2A LGALS8 CD46 TTR ARG1 FOXO3 GPAM TNFRSF10B A1BG C4BPA C4BPB MTUS1 ANXA1 TANK AHSG LYAR SNCA IQGAP2 SEC24D HK1 APOA2 AZGP1 LRP5 FLVCR1 GYG1 S100P SLC9B2 ITGA2 FABP5 POLR3D PGM2 FASN CD14 FGG FGA FGB BSG MST1 A2M BNIP3 CTNNBIP1 F2 GLRX5 PLCB1 PROS1 NCR3LG1 ENPP1 PRMT6 C2 ITGA1 GPX1 C4B_2 TXNDC5 CFB DNAJA3 SLC16A1 |
| 76 | Regulation of molecular function | MAP2K3 EPHA3 HERPUD1 PPP2R5A ATP1B3 PFN2 SCARB1 ARHGEF10L PLXNA2 CEACAM1 TESC ICAM1 TFAP4 TIMP3 FMR1 SLC39A14 MET AGFG2 GRPEL1 ARFGEF3 LOX HMGCR FAM162A PRKAR2A ERRFI1 TNFRSF10B LYPLA1 MTRR WNK4 ANXA1 PKIB TBC1D4 TANK ARRB1 AGTR1 AHSG SNCA NAF1 IQGAP2 RGS10 HMGA2 HOMER1 CAST SKI APOA2 LRP5 PLPP3 CCNYL1 SERPINI1 FABP1 PCOLCE2 ITGA2 CTHRC1 ARHGEF40 PCSK9 PDP2 GLRX RIN1 A2M CTNNBIP1 F2 PLCB1 TSPYL2 PROS1 ENPP1 ASPH DMD ITGA1 GPX1 C4B_2 PINX1 DNAJA3 SERPINF2 ASAP3 |
| 75 | Regulation of localization | MAP2K3 EPHA3 PPP2R5A ATP1B3 PFN2 ATP2B1 SCARB1 LMAN1 PLXNA2 CEACAM1 EPB41L2 APOB TESC ICAM1 TF SORBS1 SH3GLB1 MIEF1 FMR1 EEF2K ZFAND1 MET HMGCR ARSB THBS4 SLC2A1 ARG1 FOXO3 LYPLA1 ACSL3 ALKBH7 WNK4 MTUS1 ANXA1 TBC1D4 ARRB1 MPC2 AGTR1 AHSG LYAR SNCA NAF1 HOMER1 PFKM NPTN APOA2 SV2A HK2 LRP5 TPCN2 PLPP3 SLC2A2 SLC9B2 ITGA2 NDUFAF2 FABP5 NOLC1 PCSK9 CD14 MCC PPID FGG FGA FGB BSG GLRX MST1 F2 PLCB1 ENPP1 ASPH DMD C2 C4B_2 PINX1 |
| 61 | System process | SLC7A2 NPC1L1 CPS1 VIM MAP2K3 OAT ATP1B3 ATP2B1 SCARB1 CEACAM1 ICAM1 SORBS1 TIMP3 TBL1X GPR143 FMR1 SLC1A5 HMGCR PRKAR2A GLS ERRFI1 SLC2A1 SLC16A7 FOXO3 SCPEP1 WNK4 GAMT SLC6A6 SPART SLC38A2 CYP2J2 AGTR1 SNCA FOXO1 HOMER1 NPTN ATP2B2 APOA2 AZGP1 LRP5 TPCN2 SLC2A2 FABP1 ITGA2 FABP5 PGAM2 NAV2 FGG FGA FGB SNTB1 SLC29A2 CTNNBIP1 SSTR2 PLCB1 ASPH DMD ITGA1 GPX1 SERPINF2 SLC16A1 |
| 61 | Anatomical structure morphogenesis | EPHA3 IGF2BP2 PLXNA2 TPD52 CEACAM1 APOB ICAM1 PALMD MIEF1 EEF2K FGL1 MET ZBTB16 SOX6 ADGRG6 LOX THBS4 ERRFI1 LGALS8 ARG1 FOXO3 LIAS WNK4 GAMT ZNF141 SPART ANXA1 PNPT1 NAB1 LRIG3 AGTR1 GAS2 MPZL2 HMGA2 FOXO1 NPTN FMNL2 SKI HK2 LRP5 FLVCR1 POGLUT1 ITGA2 CTHRC1 NOLC1 MTSS1 FGG FGA FGB BSG MST1 BNIP3 NECTIN3 CTNNBIP1 F2 ROBO2 DMD TRIM71 ITGA1 GPX1 SERPINF2 |
| 59 | Regulation of developmental process | EPHA3 ATP2B1 PLXNA2 CEACAM1 APOB TESC ICAM1 PALMD MIEF1 ATP11C FMR1 EEF2K ZBTB16 SOX6 LOX HMGCR THBS4 ERRFI1 ADGRL2 CD46 FOXO3 GPAM GAMT SLC6A6 SPART ANXA1 PNPT1 NAB1 DUSP6 AGTR1 AHSG GAS2 HMGA2 FOXO1 NPTN FMNL2 SKI HK2 LRP5 FLVCR1 POGLUT1 SLC9B2 CTHRC1 FGG FGA FGB MST1 BNIP3 CTNNBIP1 F2 BHLHA15 PLCB1 ROBO2 ENPP1 SMOC1 PRMT6 DMD VASN SERPINF2 |
| 55 | Response to endogenous stimulus | CPS1 VIM ATP2B1 CEACAM1 APOB ICAM1 TFAP4 SORBS1 TIMP3 EEF2K SLC39A14 SOX6 HMGCS1 LOX ARSB PRKAR2A ERRFI1 SLC2A1 ARG1 FOXO3 GPAM GOT1 PPAT DDC SPART ANXA1 TBC1D4 PNPT1 AGTR1 AHSG SNCA SESN3 HMGA2 HNMT FOXO1 PELO NPTN ZFYVE9 SKI APOA2 LRP5 ITGA2 PCSK9 FGB SLC25A33 BSG AGXT SSTR2 ROBO2 ENPP1 TRIM71 PEG10 PKLR VASN |
| 50 | Immune response | VIM PAG1 CEACAM1 APOB ICAM1 TF COTL1 FGL1 TSPAN14 ALDOC ARSB PRKAR2A LGALS8 CD46 TTR ARG1 GPAM A1BG C4BPA C4BPB ANXA1 TANK AHSG LYAR SNCA IQGAP2 HK1 APOA2 GYG1 S100P FABP5 POLR3D PGM2 CD14 FGG FGA FGB A2M BNIP3 F2 PROS1 NCR3LG1 ENPP1 C2 GPX1 C4B_2 TXNDC5 CFB DNAJA3 |
| 49 | Cell population proliferation | CRLF1 SCARB1 CEACAM1 TESC TFAP4 SQLE FGL1 ZBTB16 P3H3 HMGCR THBS4 ERRFI1 CD46 ARG1 FOXO3 GPAM DAGLA ANXA1 TBRG4 ARRB1 AGTR1 TACC1 HMGA2 PELO SKI AZGP1 LRP5 ITGA2 CTHRC1 NDRG2 NOLC1 CNBP MTSS1 MCC SLC25A33 MST1 CTNNBIP1 ERN1 F2 SSTR2 PSMG1 HMGN5 DMD TRIM71 ITGA1 GPX1 PINX1 DNAJA3 SERPINF2 |
| 45 | Regulation of immune system process | PAG1 CEACAM1 APOB TESC ICAM1 ATP11C FGL1 ZBTB16 LOX THBS4 PRKAR2A CD46 ARG1 FOXO3 GPAM C4BPA C4BPB MTUS1 ANXA1 TANK LYAR SNCA HK1 APOA2 SLC9B2 ITGA2 POLR3D CD14 FGG FGA FGB MST1 A2M CTNNBIP1 F2 PLCB1 PROS1 NCR3LG1 PRMT6 C2 GPX1 C4B_2 CFB DNAJA3 |
